# Decoding the pathogenesis of Diamond–Blackfan anemia using single-cell RNA-seq

**DOI:** 10.1038/s41421-022-00389-z

**Published:** 2022-05-10

**Authors:** Bingrui Wang, Chenchen Wang, Yang Wan, Jie Gao, Yige Ma, Yingnan Zhang, Jingyuan Tong, Yingchi Zhang, Jinhua Liu, Lixian Chang, Changlu Xu, Biao Shen, Yumei Chen, Erlie Jiang, Ryo Kurita, Yukio Nakamura, Kim-Chew Lim, James Douglas Engel, Jiaxi Zhou, Tao Cheng, Xiaofan Zhu, Ping Zhu, Lihong Shi

**Affiliations:** 1 State Key Laboratory of Experimental Hematology, National Clinical Research Center for Blood Diseases, Haihe Laboratory of Cell Ecosystem, Institute of Hematology & Blood Diseases Hospital, Chinese Academy of Medical Sciences & Peking Union Medical College, Tianjin, China; 2 Department of Stem Cell and Regenerative Medicine, Peking Union Medical College, Tianjin, China; 3 Center for Stem Cell Medicine, Chinese Academy of Medical Sciences, Tianjin, China; 4 Division of Pediatric Blood Diseases Center, Institute of Hematology and Blood Diseases Hospital, Chinese Academy of Medical Sciences & Peking Union Medical College, Tianjin, China; 5 Division of Transplantation Center, Institute of Hematology and Blood Diseases Hospital, Chinese Academy of Medical Sciences & Peking Union Medical College, Tianjin, China; 6 Department of Research and Development, Central Blood Institute, Japanese Red Cross Society, Tokyo, Japan; 7 Cell Engineering Division, RIKEN BioResource Research Center, Ibaraki, Japan; 8 Department of Cell and Developmental Biology, University of Michigan Medical School, Ann Arbor, MI USA

**Keywords:** Haematopoietic stem cells, Transcriptomics

## Abstract

Ribosomal protein dysfunction causes diverse human diseases, including Diamond–Blackfan anemia (DBA). Despite the universal need for ribosomes in all cell types, the mechanisms underlying ribosomopathies, which are characterized by tissue-specific defects, are still poorly understood. In the present study, we analyzed the transcriptomes of single purified erythroid progenitors isolated from the bone marrow of DBA patients. These patients were categorized into untreated, glucocorticoid (GC)-responsive and GC-non-responsive groups. We found that erythroid progenitors from untreated DBA patients entered S-phase of the cell cycle under considerable duress, resulting in replication stress and the activation of P53 signaling. In contrast, cell cycle progression was inhibited through induction of the type 1 interferon pathway in treated, GC-responsive patients, but not in GC-non-responsive patients. Notably, a low dose of interferon alpha treatment stimulated the production of erythrocytes derived from DBA patients. By linking the innately shorter cell cycle of erythroid progenitors to DBA pathogenesis, we demonstrated that interferon-mediated cell cycle control underlies the clinical efficacy of glucocorticoids. Our study suggests that interferon administration may constitute a new alternative therapeutic strategy for the treatment of DBA. The trial was registered at www.chictr.org.cn as ChiCTR2000038510.

## Introduction

The regulation of cell proliferation and differentiation is fundamental to normal tissue and organismal growth, and deregulation of these biological processes often results in a disease state. A variety of proteins serve as pivotal players driving cellular development and differentiation, and are synthesized by organelles called ribosomes. Ribosomopathies comprise a diverse subset of disorders resulting from aberrant ribosome production, and they provide a unique opportunity to elucidate how ribosome-mediated translational control impacts normal cellular proliferation and differentiation through studying the resultant disease states. Despite significant efforts made to investigate the mechanisms controlling ribosome biosynthesis and function, there are still many unanswered questions surrounding how specific developmental anomalies are caused by ribosomal defects.

Diamond–Blackfan anemia (DBA; MIM: 105650), a rare congenital red blood cell hypoplastic disease that typically manifests in the first year of life, is one such ribosomopathy^1^. Common clinical features of DBA include anemia, macrocytic erythrocytes and elevated erythrocyte adenosine deaminase activity^2^, and the condition is often associated with short stature, physical anomalies and predisposition to cancer^3^. Previous studies indicate a notable absence of erythroblasts in patients with DBA, primarily due to a shortage of erythroid progenitors (e.g., burst-forming unit-erythroid (BFU-E) cells) in their bone marrow (BM) at the time of the initial diagnosis^4,5^, but all other hematopoietic lineages are minimally affected^6^.

The genetic basis for DBA has been extensively characterized^7,8^. Approximately 60–70% of DBA patients carry lesion(s) in genes encoding the small 40 S ribosomal protein subunit (RPS) or the large 60 S ribosomal protein subunit (RPL)^8,9^. Of the RP genes, *RPS19* is the most frequently mutated, accounting for ~25% of DBA patients^10^. In addition, mutations in non-RP genes such as the erythroid transcription factor *GATA1*^11,12^ and RPS26-interacting protein *TSR2*^13^ have also been identified. However, while the genetic causes for DBA are known, the paradox of erythroid lineage-specific defects against the background of a universal requirement for ribosomal biosynthesis in all cell types remains enigmatic.

The pathogenesis of DBA has been linked to P53 activation, which induces cell cycle arrest and cellular apoptosis of erythroid progenitors^14–17^. The liberated RPs, as a consequence of abortive ribosome assembly, cause nucleolar stress that results in P53 activation^18^. Another trigger is thought to be the accumulation of cytotoxic free heme due to the imbalance between globin protein synthesis and heme production, owing to the overall reduction of ribosome supply^19^. In addition, the compromised rate of polypeptide chain initiation will disproportionately and specifically affect mRNAs that are the least efficiently translated, for example *GATA1*^20,21^. However, despite these advances, the molecular mechanisms linking ribosomal insufficiency and defective erythropoiesis remain to be fully understood.

Glucocorticoids (GCs) and chronic red blood cell transfusion are the current standard treatment for DBA. Nearly 80% of DBA patients initially respond to GCs, but only half of these patients appear to retain long-term responsiveness^7^. GC was believed to act through increasing the proliferation of early committed erythroid progenitors^22–24^, but the mechanistic basis for the effects of GC in DBA remains unclear. Moreover, 20% of DBA patients are completely unresponsive to GC, and the resistance mechanisms are not well investigated^25^.

In this study, we conducted comparative single-cell RNA-seq (scRNA-seq) of purified erythroid progenitors isolated from the BM of DBA patients who either received no treatment or relapsed after stopping GC treatment for over 6 months (untreated, UT), or received GC treatment and responded well (GC-responsive, GCR) or poorly (GC-non-responsive, GCNR). We discovered that in DBA erythroid progenitors, elevated entry into the cell cycle is the key cause leading to the pathogenesis of DBA. GC delayed the cell cycling of erythroid progenitors of BFU-E cells by elevating type 1 interferon (IFN) signaling, a process that likely contributes to the therapeutic effects of GCs. Furthermore, IFNα treatment elevated erythrocyte production in cells derived from DBA patients. The results of our study offer a potential novel therapeutic approach for the treatment of DBA patients.

## Results

### BM erythroid progenitors in DBA patients show compromised growth

**Fig. 1:**
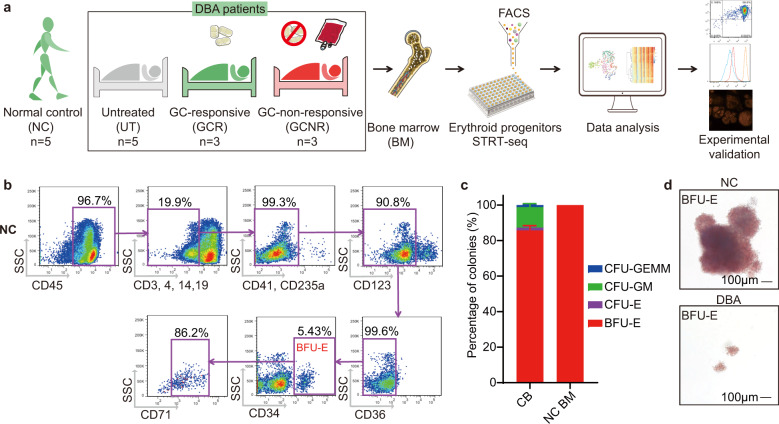
BM erythroid progenitors in DBA patients show compromised growth. **a** Schematic illustrating the experimental workflow. Bone marrow (BM) erythroid progenitors from healthy individuals (acting as normal controls, NC) and DBA patients, who were subcategorized into untreated (UT), glucocorticoid (GC)-responsive (GCR) and GC-non-responsive (GCNR) groups, were sorted using flow cytometry and processed for single-cell RNA-seq (scRNA-seq) using modified STRT-seq, followed by data analyses and experimental validation. **b** Representative FACS plots showing the gating strategies used for isolation of BFU-E cells from NCs, which were immunophenotypically defined as CD45^+^CD3^−^CD4^−^CD14^−^CD19^−^CD41^−^CD235a^−^CD123^−^CD36^−^CD34^+^. **c** The percentage of colonies generated from FACS-sorted cells (BFU-E: CD45^+^CD3^−^CD4^−^CD14^−^CD19^−^CD41^−^CD235a^−^CD123^−^CD36^−^CD34^+^) from cord blood (CB) and BM mononuclear cells. We seeded the sorted cells from the CB of three individuals and BM of one individual due to the limited healthy BM sample. Results are represented as mean ± SEM. **d** Micrographs of representative BFU-E colonies differentiated from sorted BFU-E cells of NC and DBA UT patients on day 14 of the colony forming unit assay. Scale bars, 100 μm. See also Supplementary Fig. S1, Tables S1, 2.

To investigate the mechanisms underlying DBA erythroid pathophysiology and the molecular basis underlying the therapeutic effects of GCs, we collected BM cells from patients with confirmed clinical DBA diagnoses for scRNA-seq. Of these patients, five received no treatment or relapsed after stopping GC treatment for over 6 months (untreated; UT), three showed a positive response to GC upon initial treatment (GC-responsive; GCR) and three patients failed to respond to GC upon initial treatment (GC-non-responsive; GCNR; Supplementary Table S1 and Fig. 1a). The DBA patients recruited carried mutations in various RP genes, including *RPS19*, *RPS26*, *RPL5*, *RPL11, RPL15,* and *RPL35A*. BM cells isolated from five healthy individuals were included as normal controls (NC; Supplementary Table S2).

BFU-E erythroid progenitors (CD45^+^CD3^−^CD4^−^CD14^−^CD19^−^CD41^−^CD235a^−^CD123^−^CD36^−^CD34^+^)^26^ were flow-sorted from BM mononuclear cells (BMMNCs) isolated from NCs (Fig. 1b) and patients (Supplementary Fig. S1a–c). Then, the purity of the sorted cells was experimentally validated using colony forming unit (CFU) assays. We generated >80% of BFU-E colonies in cord blood (CB)-derived cells and nearly 100% in BM-derived cells (Fig. 1c and Supplementary Fig. S1d). We also revealed that UT BFU-E progenitors formed smaller colonies than those of NC BFU-E cells (Fig. 1d). This result suggested that DBA UT erythroid progenitors have reduced proliferative potential, consistent with the findings of previous studies^5,27^.

### Erythroid progenitors in normal individuals are heterogeneous

Next, we performed scRNA-seq using a modified STRT-seq strategy^28^ to transcriptionally profile individual purified erythroid progenitors. After implementing rigorous quality control (see Materials and Methods), 426 single BFU-E erythroid progenitor cells from NCs were analyzed, with a median of 122,477 unique molecular identifiers (UMIs) and an average of 3594 genes being detected per cell (Supplementary Table S3 and Fig. S2a, b).

**Fig. 2:**
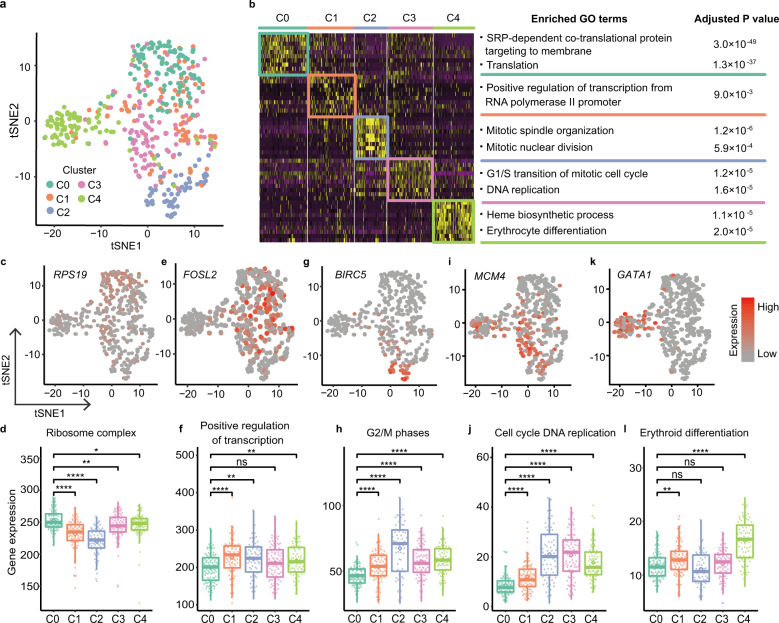
Erythroid progenitors in normal individuals are heterogeneous. **a** t-SNE visualization of distinct cell clusters of normal control (NC) BFU-E cells. **b** Heatmap (left) showing top ten marker genes and the enriched top GO terms (right) of each cluster. **c**, **e**, **g**, **i**, **k** The indicated marker genes of each cluster are color-coded and shown on t-SNE plots. **d**, **f**, **h**, **j** and **l** Beeswarm plots depict the expression of the indicated gene sets across clusters. Each dot represents the expression value of a single cell that was calculated by summing the log_2_ transformed UMI of every gene within the gene set. The diamond represents the mean expression value for each cluster, and the box represents the median and quartiles. *P* values were determined using Wilcoxon rank sum tests. ****P ≤ 0.0001, **P ≤ 0.01 and *P ≤ 0.05, ns, no significance. See also Supplementary Fig. S2 and Tables S3–S6.

Using dimension reduction and unsupervised clustering of single-cell gene expression profiles, we found that NC BFU-E cells were highly heterogeneous and could be grouped into five distinct clusters (C0-4; Fig. 2a and Supplementary Fig. S2c). To probe the biological relevance of each cluster, we identified marker genes for each cluster and conducted gene ontology (GO) enrichment analysis (Fig. 2b and Supplementary Tables S4,S5). C0 cells actively and abundantly expressed genes associated with protein translation (e.g., *RPS19*, *RPL5*, and *RPL15)* (Fig. 2c, d, Supplementary Fig. S2d and Table S6). Consistent with this finding, gene set enrichment analysis (GSEA) identified significantly enriched ribosome complexes in C0 (Supplementary Fig. S2d). Cells in C1 were characterized as transcriptionally active with high expression levels of transcription factors, including *FOSL2* (Fig. 2b, e and f). We also found that cell cycling was an important feature of BFU-E cells, consistent with results of a previous study^29^. For example, the signature genes in C2 cells participate in mitotic spindle organization and mitotic nuclear division (Fig. 2b, g, h and Supplementary Fig. S2e), indicating that C2 cells were in G2/M phase, in agreement with the GSEA results (Supplementary Fig. S2e). Accordingly, *BIRC5*^30^, which is essential for chromosome alignment and segregation (Fig. 2g), *NUF2*^31^, which is associated with centromeres and is required for chromosome segregation, and *AURKB*^32^, which encodes a kinase involved in the regulation of chromosome alignment and segregation, were all enriched in C2 (Supplementary Fig. S2e). Both GO and GSEA analyses of C3 marker genes showed enrichment in G1/S transition and DNA replication (Fig. 2b, i, j and Supplementary Fig. S2f) with representative expression of key components of the pre-replication complex *MCM4* and *MCM5*^33^, as well as *DUT*^34^, which encodes an enzyme required for thymine synthesis and thus DNA replication. Finally, GO analysis indicated that cells in C4 were poised at the onset of the erythroid differentiation program^35^ (Fig. 2b, k, l and Supplementary Fig. S2g) exhibiting induction of key erythroid regulators (*KLF1, TAL1, and GATA1*; Fig. 2k and Supplementary Fig. S2g, h), globin protein (*HBD;* Supplementary Fig. S2g) and erythroid-specific surface markers and receptors (*TFRC* (*CD71*), *CD36* and *EPOR;* Supplementary Fig. S2h). Interestingly, alongside an abrupt upregulation of GATA1 expression in C4 (Supplementary Fig. S2h), GATA2 expression was gradually downregulated from C0 to C4, indicating that a transcriptional shift was occurring between GATA1 and GATA2 in BFU-E cells (Supplementary Fig. S2i). With the onset of the erythroid differentiation program, the expression of enzymes involved in heme biosynthesis (e.g., *FECH*, *FXN, ALAD*, and *UROD*^36^) was also substantially increased in C4 cells (Supplementary Fig. S2j, k).

To further verify the onset of erythroid differentiation in C4 cells, we performed scRNA-seq analyses of the more committed erythroid progenitors, termed colony-forming unit-erythroid (CFU-E; CD45^+^CD3^−^CD4^−^CD14^−^CD19^−^CD41^−^CD235a^−^CD123^−^CD36^+^CD71^high^) in NC group (Supplementary Table S3). As expected, the global transcriptome of CFU-E cells was aligned most closely with C4 cells (Supplementary Fig. S2l). Further analyses of gene expression profiles indicated that CFU-E cells shared the highest correlation with C4, and in descending order with the other four clusters (C3–C0) (Supplementary Fig. S2m). In summary, we found that the heterogeneity in NC BFU-E erythroid progenitors reflects their distinct self-renewal, proliferation and differentiation states.

### Glucocorticoid treatment qualitatively improves the erythroid-primed C4 subpopulation by reducing apoptosis

**Fig. 3:**
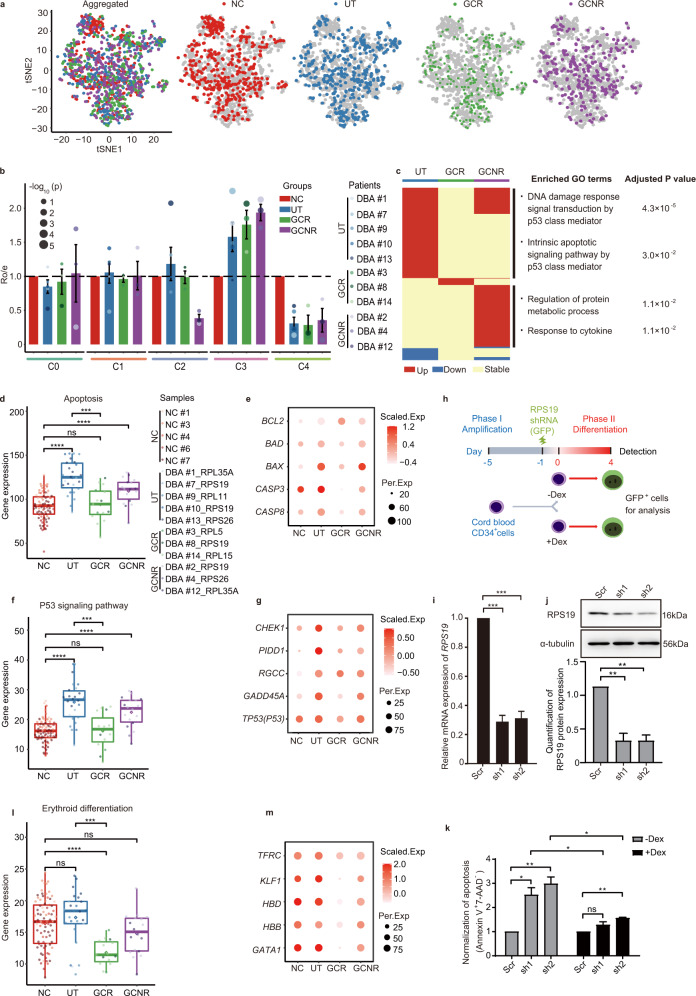
Glucocorticoid treatment qualitatively improves the erythroid-primed C4 subpopulation by reducing apoptosis. **a** t-SNE visualization of aggregated cells (Aggregated), cells from the normal controls (NC, red) and cells from the individual DBA groups (UT, dark blue; GCR, green; GCNR, purple). **b** Bar plot showing the ratio of observed to expected numbers of cells (Ro/e) in each cluster of each group (NC, red; UT, dark blue; GCR, green; GCNR, purple). The colored dots indicate the individual patients and dot size represents log_10_ transformed *P* values (Chi-square test). Error bars represent the SEM. **c** Heatmap (left) showing the differentially expressed genes during C4 in the UT, GCR and GCNR groups compared with the NC counterparts. Upregulated genes, downregulated genes and genes without significant changes are indicated in red, blue and yellow, respectively. The right panel shows the enriched GO terms between NC and UT or GCNR. **d** Beeswarm plots depict the expression of the gene set of apoptosis. The controls and patients in each group are presented and have been individually color-coded. **e** Dot plots of representative apoptosis-associated genes across groups. The scaled expression value (Scaled.Exp) and percentage of expressing cells (Per.Exp) from each group are depicted. **f** Expression of the entire gene set of the P53 signaling pathway. **g** Dot plots of representative genes in the P53 signaling pathway across groups. The scaled expression value and percentage of expressing cells from each group are depicted. **h** Schematic illustrating the two-phase erythroid differentiation culture and lentiviral shRNA vector (containing a GFP expression cassette) infection of cord blood CD34^+^ HSPCs. Four days after initiation of the 2nd differentiation phase, GFP^+^ cells were sorted for further analyses. **i** The level of *RPS19* mRNA detected using RT-qPCR in GFP^+^ cells infected with scrambled control (Scr) or RPS19 shRNA lentivirus (sh1 or sh2). *ACTB* serves as the internal control. **j** The upper panel shows RPS19 protein expression detected using Western blot in GFP^+^ cells infected with scrambled control (Scr) or RPS19 shRNA lentivirus (sh1 or sh2). α-tubulin serves as the loading control. The bottom panel indicates the corresponding quantification of RPS19 protein expression. **k** Bar graph showing the apoptotic level (measured by the percentage Annexin V^+^7-AAD^−^ cells) in RPS19-depleted erythroid cells cultured with or without Dex, which was normalized to the scramble (Scr) control. **l** The expression of the entire gene set of erythroid differentiation across groups. **m** Dot plots of representative genes related to erythroid differentiation across groups. The scaled expression value and percentage of expressing cells from each group are depicted. For the bar graphs (**i**, **j**, **k**), results are presented as the mean ± SEM. *P* values were determined by Student’s *t* test. ***P < 0.001, **P < 0.01 and *P < 0.05; ns, no significance. *n* ≥ 3 independent experiments. For all the beeswarm plots (**d**, **f**, and **l**), the controls and patients in each group are presented and color-coded individually. Each dot represents the expression value for each single cell that was calculated by summing the log_2_ transformed UMI of every gene within the gene set. Diamonds represent mean expression values for each cluster, and boxes represent the median and quartiles. *P* values were determined by Wilcoxon rank sum test. ****P ≤ 0.0001 and ***P ≤ 0.001, ns, no significance. See also Supplementary Figs. S3, 4 and Supplementary Table S6.

Next, we investigated the cellular and molecular alterations by integrating all single BFU-E cells. After applying quality control metrics, we proceeded with 1392 cells, comprised of 426, 428, 276, and 262 cells from NC, UT, GCR, and GCNR patients, respectively (Supplementary Table S3). All five cell clusters identified in NC were also present in the UT, GCR and GCNR DBA samples (Fig. 3a and Supplementary Fig. S3a–c). Furthermore, patients with different RP genetic lesions displayed similar clustering patterns and high correlations among their gene expression profiles (Supplementary Fig. S3c, d). Thus, cluster-based analyses were subsequently performed on the UT, GCR and GCNR groups regardless of the specific RP gene mutations present in the patients.

We initially determined the frequency of cells (i.e., the ratio of observed cell number to the expected cell number, Ro/e) for each cluster by implementing Chi-Squared test^37^. It is notable that DBA patients showed significantly higher C3 frequency, but a decreased C4 frequency (Fig. 3b), suggesting that erythroid development was blocked at C3 in DBA patients. The frequency of other clusters was comparable among all groups with the exception of the C2 population, which was markedly diminished in GCNR patients (Fig. 3b).

Next, we performed GO enrichment analysis of differentially expressed genes (DEGs) within each cluster between the control and DBA cells (Supplementary Fig. S4a–d). In UT C4 cells, genes associated with apoptosis and P53 signaling pathway were significantly enriched (Fig. 3c, d, f and Supplementary Fig. S4d), concordant with the GSEA results (Supplementary Fig. S4e, f). Consequently, pro-apoptotic genes, such as *BAX*^38^ and *BAD*^39,40^ (Fig. 3e), and direct effectors in P53 pathway, such as *PIDD1*, *RGCC*, and *GADD45A*^41^ (Fig. 3g), were significantly induced in UT C4 cells.

In contrast, the overall transcriptomics of C4 cells from GCR patients closely resembled that of the NC group (Fig. 3c colored with yellow). In GCR patients, but not GCNR patients, apoptosis was markedly decreased when compared to UT cells (Fig. 3d and Supplementary Fig. S4e). This was supported by a substantial downregulation of pro-apoptotic genes (*BAX* and *BAD*) and significant upregulation of the anti-apoptotic gene *BCL2*^40^ (Fig. 3e). A similar trend was observed in the P53 pathway in GCR C4 cells (Fig. 3f, g and Supplementary Fig. S4f). Since the frequency of C4 cells was similarly low in UT and GCR patients (Fig. 3b), it is tempting to speculate that GC may confer its protective effects by counterbalancing the P53 and apoptotic pathways, thereby improving the intrinsic quality of the cells and boosting their survival.

To ascertain whether apoptosis was enhanced in RP-mutant cells but reduced after GC treatment, we used a two-phase culture system to differentiate CB-CD34^+^ hematopoietic stem and progenitor cells (HSPCs) along the erythroid lineage ex vivo (Fig. 3h). After 4 days of cell expansion (phase I), we infected these primary erythroid cells with lentiviral mediated-*RPS19* shRNAs (sh1 and sh2) or a scrambled control (Scr) expressing GFP. Cells were then differentiated in the presence or absence of the synthetic corticosteroid dexamethasone (Dex, 1 μM) from days 0 to 4 in the 2nd phase. Successful reduction of RPS19 abundance was confirmed at both the mRNA (~70% reduction) (Fig. 3i) and protein level (Fig. 3j) in FACS-sorted GFP^+^ cells after 4 days of differentiation. When we examined the apoptotic indices of GFP^+^ cells with Annexin V and 7-aminoactinomycin D (7-AAD) staining, we observed that the early apoptosis index (Annexin V^+^7-AAD^−^) was high in the RPS19-deficient cells, but significantly reduced after Dex treatment (Fig. 3k). Furthermore, we also examined apoptosis level in *RPS19* heterozygous-deleted HUDEP-2 cells (*RPS19*^+/−^ cells lines: sg3-4 and sg3-8), which were generated by using CRISPR/Cas9 technology (Supplementary Fig. S4g, h). HUDEP-2 cells are an immortalized human erythroid progenitor cell line^42^. We consistently found that *RPS19* haploinsufficiency induced apoptosis while Dex treatment reduced it (Supplementary Fig. S4i, j).

In addition, we noted that the expression of genes associated with erythroid differentiation were decreased in C4 cells from GCR patients compared with the NC group (Fig. 3l and Supplementary Fig. S4k), reflecting erythroid differentiation was severely delayed in C4 cells. For example, there was hardly any detectable expression of erythroid-specific genes (including *GATA1*, *KLF1*, and *HBB*) in GCR cells (Fig. 3m). Accordingly, the GATA1 targets^20^ were significantly downregulated in C4 GCR cells compared to UT (Supplementary Fig. S4l–o and Supplementary Table S6). In addition, the expression of genes related with heme biosynthesis and globin production was also significantly reduced in C4 GCR cells compared with NC cells (Supplementary Fig. S4p, q).

Taken together, our results suggest that ribosome haploinsufficiency leads to activation of the P53 pathway and to apoptosis in erythroid progenitor cells of UT DBA patients. Furthermore, GC treatment considerably dampens P53 activation, thereby suppressing the apoptotic response. This sufficiently improves the quality of the erythroid-primed C4 subpopulation, which may enhance the survival of cells from GCR patients.

### Untreated DBA erythroid progenitor cells are forced to progress into the cell cycle

**Fig. 4:**
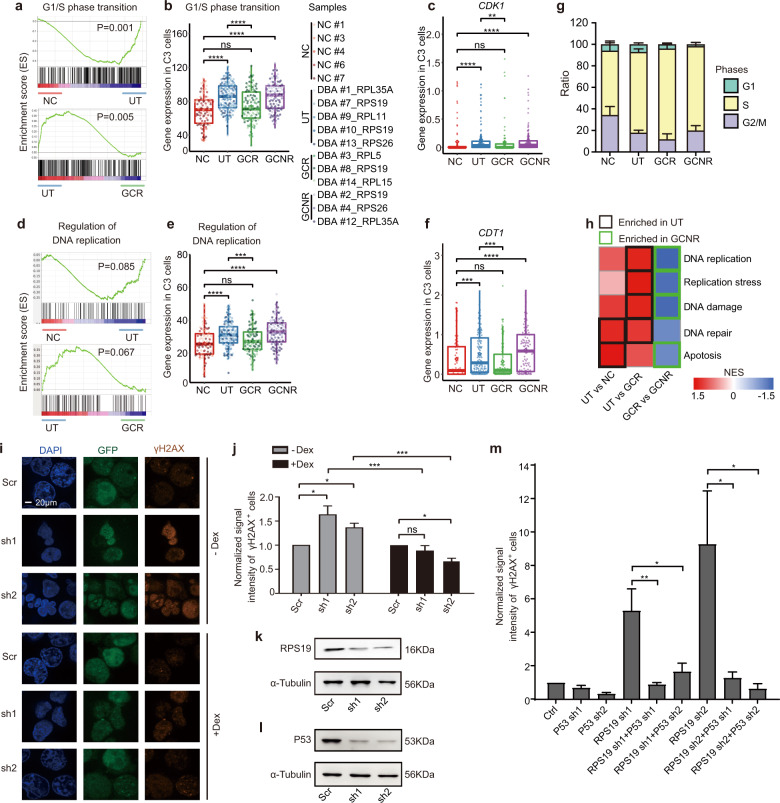
Untreated DBA erythroid progenitor cells are forced to progress into the cell cycle. **a** GSEA of G1/S phase transition in C4 between the NC and UT, and the UT and GCR groups. **b** Beeswarm plot showing the expression of the G1/S phase transition gene set in C3 among the samples. **c** *CDK1* expression in C3 is shown among groups. **d** GSEA of regulation of DNA replication between the NC and UT and the UT and GCR groups. **e** Beeswarm plot showing the expression of the DNA replication gene set in C3 among the samples. **f** The expression of *CDT1* in C3 is shown among groups. **g** The fraction of cells in G1, S and G2/M in C3 among groups. **h** Heatmap representing normalized enrichment score (NES) from GSEA for comparison between the indicated groups in C3 cells at the S-phase of cell cycle; the colored rectangles indicate significant enrichment (namely, higher expression) of corresponding processes in the designated groups (*P* < 0.05). **i** Immunofluorescence measuring DNA damage with γH2AX expression in *RPS19*-diminished primary erythroid cells on day 4 of differentiation with or without Dex treatment. **j** Quantification of immunofluorescent intensity of γH2AX. The signal intensity of *RPS19*- depleted cells was normalized to the scramble (Scr) control. **k**, **l** Western blot analysis showing the protein expression of RPS19 (**k**) and P53 (**l**) after lentiviral- mediated shRNA knockdown in erythroid cells derived from CB-CD34^+^ cells on day 8 of differentiation. α-tubulin serves as the loading control. *n* = 3 independent experiments. **m** Quantification of immunofluorescent intensity of γH2AX in erythroid cells derived from CB-CD34^+^ cells on day 8 of differentiation. The signal intensity of each group (*RPS19* single, P53 single or their double depleted cells) was normalized to the corresponding control. For (**j** and **m**), results are presented as the mean ± SEM. *P* values were determined using Student’s *t* test, ***P < 0.001; *P < 0.05;ns, no statistical significance. *n* ≥ 3 independent experiments. For all the beeswarm plots (**b**, **c**, **e** and **f**), each dot represents the expression value for each single cell that was calculated by summing the log_2_ transformed UMI of every gene within the gene set. Diamonds represent mean expression values for each cluster, and boxes represent the median and quartiles. *P* values were determined using Wilcoxon rank sum tests. ****P ≤ 0.0001, ***P ≤ 0.001 and **P ≤ 0.01; ns, no significance. See also Supplementary Figs. S5,6 and Table S6.

To elucidate the mechanistic basis of the increased apoptosis and P53 signaling observed in erythroid progenitors of UT patients, we examined C3 cells where the erythroid developmental trajectory appeared to be arrested (Fig. 3b). Given that C3 cells were characterized as being at the G1/S transition, with substantial DNA replication occurring (Fig. 2b, i, j and Supplementary Fig. S2f), we conducted GSEA with the G1/S transition gene set and compared NC and UT cells, and then UT and GCR cells (Fig. 4a). Remarkably, we found that the expression of genes associated with the G1/S transition was significantly increased in the UT group (NC vs UT), but was remarkably reduced in the GCR group (GCR vs UT) (Fig. 4a, b). Key regulators and mediators essential for promoting G1/S conversion, such as *CDK1*, *CDC25A*, and *CCND3*^43,44^, were induced in UT cells (Fig. 4c and Supplementary Fig. S5a), reflecting active S-phase entry. This was further underscored by cell cycle analysis. Compared with NC cells, more C3 cells from UT patients were in S-phase of the cell cycle (Fig. 4g). Consistently, the DNA replication program was engaged (Fig. 4d, e), with enhanced expression of replication licensing factor *CDT1* (Fig. 4f), single strand DNA binding protein *RPA1* and a component of the replication focal center *WRN*^45–47^ in UT C3 cells (Supplementary Fig. S5b). Therefore, scRNA-seq analysis of untreated DBA patient-derived cells showed that these erythroid progenitors were propelled into S-phase of the cell cycle in order to meet the daily need for red blood cell production.

Previous studies have demonstrated that erythroid progenitors display a unique S-phase-dependent cell fate switch, during which less mature erythroid progenitors transit from a self-renewal state into a phase of initial active erythroid gene transcription^29,48^. This S-phase is shorter and faster, with increased DNA replication fork speed^49^. We therefore postulated that the imbalance between rapid DNA replication and insufficient protein synthesis attributed to RP haploinsufficiency might trigger replication stress^50^, leading to DNA damage in DBA erythroid progenitors. To test this hypothesis, we assayed the processes of DNA replication, DNA replication stress, DNA damage and DNA repair in C3 cells that were in S-phase (Fig. 4h and Supplementary Fig. S5c–n). Compared with NC cells, DNA replication was hyperactivated in UT cells, as reflected by the increased abundance of the replication initiation effectors *MCM7*, *MCM5,* and DNA ligase I (*LIG1*) (Supplementary Fig. S5c–e). With inadequate protein synthetic machinery, replication stress then occurred (Supplementary Fig. S5f–h), indicated by the upregulation of genes associated with the replication stress response^50^. These genes included *RAD1*^51^, which belongs to a protein complex activated in response to incomplete DNA replication, and *ORC6*^52^, which is involved in forming a platform to assemble additional replication initiation factors. In addition, expression of the DNA damage responder *ATAD5* and mediator *PARP1*^53^ was induced (Supplementary Fig. S5i–k), along with genes that modulate the DNA repair process (such as *POLE2, POLE3* and *TIMELESS*^53^) (Supplementary Fig. S5l–n). Consequently, as anticipated, we detected elevated pro-apoptotic gene expression (*BAX* and *CASP8*) in UT cells at S-phase (Supplementary Fig. S5o–q). In contrast to the UT cells, the same processes in the GCR group were significantly downregulated, to levels closely resembling those in NC cells (Fig. 4h and Supplementary Fig. S5c–q). Therefore, it seems that in UT cells DNA replication stress activated the DNA damage response and apoptosis, whereas GC treatment reduced DNA damage and inhibited apoptosis by alleviating DNA replication stress in GCR cells.

To investigate whether GC relieves DBA replication stress and DNA damage, Dex-treated or untreated GFP^+^ erythroid cells from CB-derived CD34^+^ HSPCs were collected on day 4 of differentiation for DNA damage analysis using an anti-γH2AX immunofluorescence assay. We observed increased DNA damage, as reflected by anti-γH2AX immune-positivity in the RPS19-deficient cells that was alleviated when the same cells were cultured with Dex (Fig. 4i, j). A similar trend of reduced DNA damage was also found in the *RPS19*^+/−^ cells treated with Dex (Supplementary Fig. S6a, b).

Furthermore, to examine the potential link between P53 activation and DNA damage, we infected CB-derived CD34^+^ HSPCs with either *RPS19* shRNAs expressing GFP or *P53* shRNAs expressing puromycin, alone or in their combination, on day 4 of phase I proliferation (Fig. 3h). During the induction of phase II erythroid differentiation, RPS19 and P53 expression levels were examined on day 8 of differentiation by western blot (Fig. 4k, l). DNA damage levels were significantly reduced in the *RPS19* and *P53* double-depleted cells compared with *RPS19* single-depleted cells (Fig. 4m and Supplementary Fig. S6c).

In summary, rather than cell cycle arrest^54,55^, we found that DBA patient UT progenitor cells are forcibly pushed into the cell cycle. However, the contradiction between the innately fast cell cycle property of enhanced DNA replication in erythroid progenitors and inadequate protein synthesis could possibly trigger P53 activation. This may be one of the key mechanisms underlying the pathogenesis of DBA.

### Glucocorticoid treatment attenuates cell proliferation by elevating IFN signaling

**Fig. 5:**
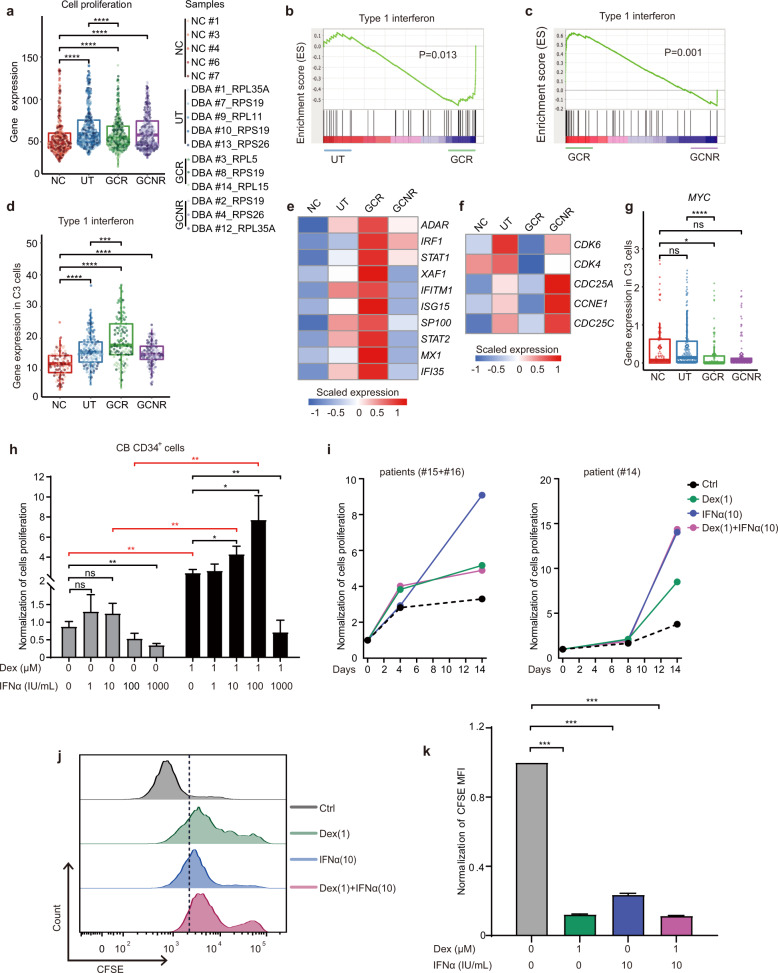
Glucocorticoid treatment attenuates cell proliferation by elevating IFN signaling. **a** Proliferation scores^56^ were estimated for all groups. **b**, **c** GSEA of type 1 interferon in C3 cells comparing UT to GCR (**b**) and GCR to GCNR (**c**) groups. **d** Beeswarm plot showing the expression of type 1 interferon in C3 among different groups. **e** Heatmap illustrating scaled expression of key genes in the type 1 interferon signaling pathway in C3 cells from the NC, UT, GCR and GCNR groups. **f** Heatmap showing scaled expression of cell cycle mediators in C3 of NC, UT, GCR and GCNR groups. **g** The expression of *MYC* in C3 is shown among groups. **h** The proliferation of *RPS19-*depleted, cord blood-derived erythroid cells treated with either IFNα or Dex alone, or their combination, assessed on day 4 of erythroid differentiation. Proliferation was normalized to the non-treated control cells. **i** Time course growth curves of patient-derived BM-CD34^+^ cells during erythroid differentiation, cultured either with Dex or IFNα alone, or their combination. Due to the limited cells available from the patients, we occasionally combined the patient samples together. **j** Representative FACS plots of CFSE staining in GCR patient-derived BM-CD34^+^ cells cultured either with Dex or IFNα alone or in their combination on day 8 of erythroid differentiation. **k** Quantification of (**j**). The MFI of each group of cells was normalized to that of non-treated control cells. For (**h** and **k**), the error bars represent the SEM and *P* values were determined using Student’s *t* tests. ***P < 0.001; **P < 0.01 and *P < 0.05. *n* = 3 biological replicates. For the beeswarm plots (**a**, **d** and **g**), each dot represents the expression value that was calculated by summing the log_2_ transformed UMI of every gene within the gene set of a single cell. Diamonds represent mean expression values for each cluster, and boxes represent the median and quartiles. *P* values were determined using Wilcoxon rank sum tests. ****P ≤ 0.0001, ***P ≤ 0.001 and *P ≤ 0.05; ns, no significance. See also Supplementary Fig. S7 and Table S6.

We noted excessive DNA replication, damage and repair in S-phase of UT DBA patient cells, and these properties reverted to normal levels in GCR cells (Supplementary Fig. S5c–n). However, the fraction of cells in the G1, S and G2/M phases was similar between the UT and GCR groups (Fig. 4g). This suggested that, rather than the fraction of cells, it might be the cell cycle per se (e.g., duration) that is altered in GCR cells. To investigate this hypothesis, we scored the proliferative potential (an indicator of cell cycle duration) of individual cells by pooling the expression of module genes indicative of active cell amplification^56^. We found that cells from UT and GCNR groups exhibited higher proliferative scores compared with the GCR and NC groups (Fig. 5a and Supplementary Fig. S7a). We then speculated that cells from the UT and GCNR groups might remain in the more rapid cell cycling mode with a higher proliferative index. In contrast, GCR cells might have a lower proliferative potential as a result of decreasing proliferative signature gene expression.

Next, to delve into the underlying molecular basis of the altered proliferation in GCR cells, we examined GO analysis of DEGs between the control and DBA C3 cells (Supplementary Fig. S4c). Intriguingly, the top enriched GO terms included the type 1 interferon signaling pathway, which was strongly expressed in cells from GCR patients when compared with cells from NC patients (*P* = 4.6 × 10^−6^, Supplementary Fig. S4c). This result was consistent with GSEA and expression of associated gene sets (Fig. 5b–d). As shown in Fig. 5e, key components of the IFN signaling pathway, such as *STAT1*, *STAT2*, *IRF1*, *MX1* and *ISG15*, were upregulated in GCR C3 cells, but not GCNR C3 cells (Fig. 5e).

Accumulating evidence has demonstrated that IFN signaling exerts anti-proliferative effects in a variety of cell types by extending the cell cycle through prolongation of the S phase and preventing the G1/S transition^57,58^. To understand how IFN signaling affects the proliferation of GCR cells, we profiled cell cycle mediators and effectors, such as *CDK4*, *CDK6*, *CDC25A*, *CDC25C*, and *CCNE1*, and found that their expression was lower than those in UT cells (Fig. 5f and Supplementary Fig. S7c, d). Previous studies have shown that IFN-mediated cell cycle delay is associated with downregulation of *MYC*^59^, which promotes cell proliferation by stimulating the cell cycle^60^, and we detected that *MYC* was also downregulated in GCR cells (Fig. 5g). Thus, it seems likely that GC treatment delays cell cycle progression and attenuates cell proliferation by increasing the activity of the IFN pathway. When cell proliferation was reduced, the imbalance between intrinsically rapid cell proliferation and insufficient protein synthesis was reconciled, avoiding P53 activation and thus improving the survival of DBA BFU-E cells.

To experimentally determine whether IFN signaling affects the growth properties of RP-deficient erythroid progenitors, IFNα (1, 10, 100, and 1000 IU/mL) was added to the culture media of *RPS19*-reduced, CB-derived CD34^+^ cells from the initiation of differentiation, with or without Dex (1 μM) treatment. We found that, compared with the untreated control, a low dosage of IFNα (1 and 10 IU/mL) significantly induced cellular output on day 5 of erythroid differentiation (Supplementary Fig. S7d). Of note, a combination of Dex and IFNα treatment achieved a synergistic effect during erythroid differentiation in these cells on day 4 (Fig. 5h) and 5 of differentiation (Supplementary Fig. S7d).

Encouraged by this preliminary observation, we further tested such potential stimulatory effects on patient-derived erythroid cells. To this end, we harvested CD34^+^ HSPCs from the BM of DBA patients and induced ex vivo erythroid differentiation. The CD34^+^ cells were collected from five DBA patients (Supplementary Table S1), who turned out to be GC-responsive patients by the follow-up studies. During the induction of erythroid differentiation, cells were treated either with Dex or IFNα alone or in combination from day 0 to 14. On day 14, we found that IFNα treatment yielded more erythroid cells than untreated controls (Fig. 5i). To assess the impact of such treatments on the cell cycle, we conducted CFSE assays and uncovered that cell cycle progression was indeed delayed in cells treated either with Dex or IFNα alone, or in combination (Fig. 5j, k). Taken together, IFNα treatment could serve as a promising alternative therapeutic strategy for DBA patients, and the combination of IFNα and Dex treatment might potentially achieve an even greater clinical efficacy.

### Glucocorticoid alleviates nucleolar stress in BFU-E cells of DBA patients

**Fig. 6:**
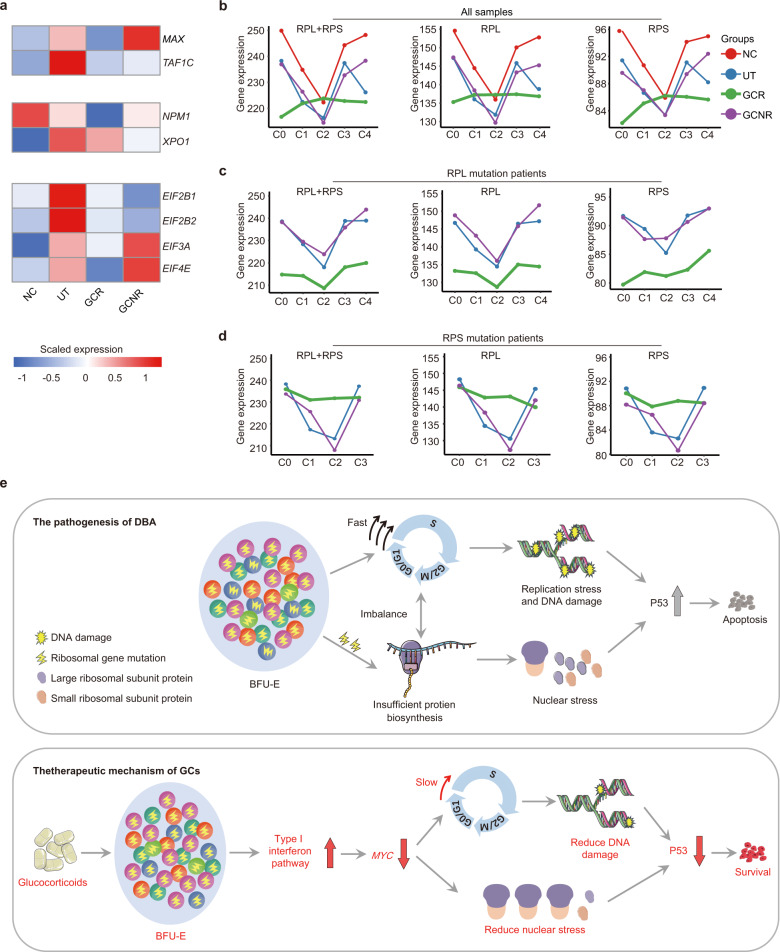
Reduced ribosomal stress attributes to the protective effect of glucocorticoids. **a** Heatmap showing the scaled expression of key regulators and/or effectors associated with ribosomal transcription (*MAX* and *TAF1C*), ribosomal processing and transporter (*NPM1* and *XPO1*) and translation initiator (*EIF2B1*, *EIF2B2*, *EIF3A*, and *EIF4E*) in C3 of all groups. **b**–**d** Line graphs showing the expression dynamics of total ribosomal protein genes (RPL + RPS), large ribosomal protein genes (RPL) and small ribosomal protein genes (RPS) in all (**b**), RPL mutant (**c**) and RPS mutant (**d**) samples across clusters. Different donor types are coded with the indicated color. **e** A hypothetical model illustrating altered biological processes that lead to cellular distress and pathogenesis of DBA (upper). The imbalance between the innate fast cell cycle and insufficient protein biosynthesis, which results from RP mutation, could trigger DNA replication stress in the BFU-E cells of DBA patients; and concomitantly elicit DNA damage-induced P53 activation and apoptosis. The lower panel shows a proposed schematic of the therapeutic mechanism of GCs. GC administration elevates IFN signaling, which attenuates cell proliferation by regulating the activity of cell cycle regulators and modulators (e.g., *MYC*, *CDK4*, and *CDK6*). It also alleviates ribosomal stress via the repression of *MYC*, thereby rectifying the imbalance and ultimately ameliorating apoptosis and promoting cell survival.

Among the targets of IFN, *MYC* was remarkably reduced in GCR BFU-E cells (Fig. 5g). Apart from accelerating cell amplification^60^, MYC is also a direct regulator of ribosome biosynthesis^61^. MYC not only regulates the transcription of ribosomal RNAs (rRNAs) and proteins, but also modulates the expression of auxiliary factors required for rRNA processing, ribosomal assembly and the export of mature ribosomal subunits. We found that, in addition to *MYC*, the expression of *MAX* and *TAF1C*, which interact with MYC to modulate rDNA and RP transcription^61^, was also reduced in the GCR group (Fig. 6a). Following this observation, we speculated that the transcription of RP and rRNA might also be reduced. When we examined the transcripts of all ribosomal protein components across clusters of control and DBA samples, we found that, in general, the expression of ribosomal components (RPL + RPS) was greatly reduced in GCR patients when compared with other groups (Fig. 6b). More interestingly, the dynamic expression pattern of these ribosomal protein components disappeared and was replaced by a relatively constant level of expression (Fig. 6b). This pattern remained unchanged even when the expression of RPL and RPS subunits within these samples was evaluated separately (Fig. 6b). Consistent with this finding, expression of genes related to ribosomal rRNA processing and ribosomal export (e.g., *NPM1*, *XPO1*) and even translation initiation (*EIF2B1, EIF2B2, EIF3A, EIF4E*) was accordingly reduced (Fig. 6a). Next, we classified DBA patients based on RPL (Fig. 6c) and RPS (Fig. 6d) mutations under distinct treatment conditions, and found a reduced, constant expression pattern of ribosome genes. Therefore, we hypothesize that reduced and/or balanced RP gene expression may alleviate the accumulation of free RPs in GCR cells (Fig. 6b–d). Excessive free RPs, arising from abortive ribosomal assembly of RP haploinsufficiency, are well-documented to elicit nucleolar stress and stabilize P53 via interaction with MDM2^62^. We thus propose that one of the molecular mechanisms underlying the efficacy of GCs could account for the reduced but balanced ribosome constituents, and serve to ameliorate nucleolar stress in DBA erythroid progenitors.

Collectively, our results suggest that GC simultaneously resets cell cycle progression and neutralizes nucleolar stress by increasing IFN signaling in the erythroid progenitor cells of DBA patients. Consequently, GC treatment promotes erythroid cell survival and thus improves the anemia induced by this disease (Fig. 6e).

## Discussion

Ribosomopathies, often manifesting as specific cell and tissue defects, are commonly caused by ribosomal protein haploinsufficiency or defects in ribosome biogenesis. The tissue specificity of these disorders with the ubiquitous requirement for ribosomes in all cells is of fundamental biological interest and of clinical relevance. In this study, using DBA as a model, we revealed the innately shorter cell cycle of erythroid progenitors underlay DBA pathogenesis and thus linked cell cycle process to translational apparatus and lineage differentiation.

In a healthy adult, about 200 billion of erythrocytes are produced daily. This requires exponential expansion of committed erythroid progenitors. In our current study, at single cell resolution, we showed that proliferation and differentiation program were associated with distinct progenitor subpopulation. The immature GATA1^−^ BFU-E cells mainly undergo self-renewal and proliferation and are characterized by high expression of marker genes correlated with cell cycle progression (C2 and C3) and active protein translation (C0). These results imply that the immature BFU-E cells are responsible for producing a vast number of erythrocytes with the reserving of sufficient translational machinery. Following the continuum of erythroid developmental trajectory^63^, the committal step of the onset of erythroid program was detected in mature BFU-E subpopulation, coinciding with the activation of erythroid master regulator GATA1 (GATA1^+^ BFU-E)^64^ as well as the initiation of globin transcription. Here, utilizing scRNA-seq analysis of the heterogeneous normal BM BFU-E progenitors presented as the reference point, we performed comparative global transcriptomic profiling of BFU-E cells from DBA patients to molecularly probe the basis for DBA pathogenesis.

We delineated a number of aberrant molecular events that gave rise to the pathogenesis of DBA. For example, the erythroid progenitors in C3 cells were forcibly propelled into cell cycle via overactivation of the cyclin D complex (*CCND3*/*CDK4*/*6*). The cyclin D complex can phosphorylate and inactivate the retinoblastoma protein RB1, thereby facilitating the entry and progression of cells into S-phase^44^. Elegant studies have demonstrated that erythroid progenitors have unique cell cycle signatures with shorter cell cycle duration and faster DNA synthesis during the commitment from self-renewal to differentiation^29,49^. Such a highly active S-phase has been shown to be sensitive to other cues or stresses^49^. Thus, upon encountering an insufficient protein supply, DBA patient-derived progenitor cells at S-phase suffer constitutive replication stress, which induces DNA damage and P53 activation, as indicated by the data presented here. Therefore, it is highly possible that because BFU-E progenitors are forced into active cycling, the contradiction between the inherently faster cell cycle and DNA replication coupled with a limited protein supply could reflect the molecular basis for the pathogenesis of DBA. These in vivo findings challenge the results of previous studies, which were primarily conducted in vitro, and suggested that erythroid cells are arrested at G1 stage due to P53 activation^65–67^. This discrepancy could be explained by a negative feedback loop in the critical demand for erythrocytes which is caused by anemia in DBA patients.

The pathogenesis of DBA has also been linked to *GATA1*, given that *GATA1* mutation has been identified in DBA patients and its translation is impaired by ribosome mutations; largely due to the complex structure of *GATA1* mRNA^11,20,21^. In the present study, we detected the onset of erythroid defects in erythroid progenitors as early as the *GATA1*^−^ immature BFU-E C3 subset. However, even in *GATA1*^+^ C4 cells, we found that the expression of GATA1 targets^20^ was elevated in UT patients. These results suggested that inefficient translation of GATA1 might not significantly impact the activation of GATA1 targets in the BFU-E cells of RP-mutant UT patients. Consistent with these results, previous studies have demonstrated that GATA1 deletion does not markedly alter erythroid commitment, but blocks erythroid differentiation primarily at the proerythroblast stage^68,69^. We therefore hypothesize that ribosome haploinsufficiency leads to replication stress and triggers apoptosis in the early self-renewing *GATA1*^−^ BFU-E C3 subpopulation, and inefficient translation of *GATA1* further exacerbates anemia in DBA patients during terminal erythroid differentiation.

At present, GCs are the first-line treatment strategy for DBA. Despite decades of clinical administration, the molecular understanding of GC efficacy in DBA patients remains incomplete. Recently, Li et al. demonstrated that, rather than directly stimulating self-renewal through cell cycle alteration in normal erythroid progenitors^23,24^, GC extends and delays the commitment process^63^, thereby permitting more cell divisions prior to terminal differentiation. Under pathological conditions, we also observed delayed progression into the erythroid differentiation program in GCR patient cells. In addition, we found that cell proliferation potential was diminished in GCR patient cells; a result that could be attributed to the activation of IFN signaling. IFN signaling is well-documented to exert direct anti-proliferative effects on a variety of cells, including erythroid progenitor cells (BFU-E and CFU-E)^70,71^. Such effects are mediated via multifactorial mechanisms^57,58^, including prolonging the cell cycle by suppression of S-phase DNA synthesis^72,73^ and inhibiting G1/S transition via suppression of the G1 CDK-complex^74,75^. In addition to cell cycle regulation, IFN exerts regulatory functions in nucleolar stress, partially through its direct target *MYC*^59,76^. Therefore, the activation of IFN signaling appears to contribute to the clinical benefit of GCs by coordinating cell cycle regulation and nuclear stress mediation. In summary, it is likely that, in UT-DBA patients, the C3 progenitors are forced into active cycling. Then the contradiction between the innately faster cell cycle and a paucity of protein supply provokes the DNA damage and upregulates P53 signaling pathway, which ultimately induces the apoptosis of C4 cells. Conversely, in GCR group, GC treatment attenuates C3 cell proliferation and extends the cell cycle by elevating IFN signaling. When cell proliferation is reduced, the imbalance between intrinsically rapid cell proliferation and insufficient protein synthesis can be reconciled, avoiding P53 activation and thus improving the survival of C4 cells.

We also noted that the IFN signaling was increased in UT patients, which is in line with previous observations in zebrafish with DBA manifestation and in *RPS19*-deficient erythroid cells derived from human CD34^+^ cells^77^. We speculated that such activation of the IFNα response in the cells of UT patients could represent a feedback mechanism attempting to sustain cell survival. However, since the increased IFN levels are not sufficient (as compared with the GCR group), a substantial number of BFU-E cells continued to undergo apoptosis. In addition, although a recent study demonstrated that GC stimulated the proliferation of *RPS19*-deficient CFU-E progenitors by upregulating *P57*^*KIP2*^(*CDKN1C*)^78^, we found that the effects of GC on DBA BFU-E cells were independent of *P57*^*KIP2*^, as its expression was nearly undetectable at this stage. Unfortunately, we failed to collect CFU-E cells from patients in the present study and therefore did not have a chance to compare the expression of *P57*^*KIP2*^ in GCR and GCNR patients. Given that the expression of *P57*^*KIP2*^ was undetectable in BFU-E cells, one possible interpretation could be that GCs exert stage-specific effects on BFU-E and CFU-E cells; a hypothesis that awaits future investigation.

To date, 20% of DBA patients do not respond to GC treatment, and the reasons for this are unknown^25^. Even in the 80% of patients who initially respond to GCs, half of them become unresponsive to GC treatment over time. The only definitive curative approach is allogeneic hematopoietic stem cell transplantation, which carries the risk of adverse events and mortality remains significant. Thus, a safe and efficient therapeutic approach for the treatment of DBA patients remains wanting. An alternative strategy could benefit patients who are unresponsive to GCs or patients who are responsive to GCs but suffer from steroid toxicity, experiencing osteonecrosis of the femoral head, cataract or avascular necrosis^79^. In the present study, we found that IFNα treatment alone promoted the generation of erythroid cells in both RPS19-reduced primary erythroid cells and BM cells collected from DBA patients, whose erythroid cells were differentiated ex vivo. Given that the safety of IFN treatment in children is well-documented, as it has been widely used to treat chronic hepatitis B in this age group^80^, IFNα could represent a potential alternative therapy for DBA. More notably, we found that Dex and IFNα treatment produced a synergistic effect on erythroid production during ex vivo induction of erythroid differentiation of BM-CD34^+^ cells, suggesting that a combined therapeutic approach might achieve better clinical outcomes. However, further studies using a larger patient cohort are needed.

One additional limitation faced by the present study was that, due to the young age of the patients when BM samples were collected (median 15 months old), it is difficult to obtain BM cells from age-matched controls and harvest sufficient erythroid progenitors for proteomic study. In the future, to further clarify the pathogenesis of DBA, this avenue of investigation should be pursued using newly-developed proteomic strategies with more suitable controls that have greater sensitivity.

In summary, by analyzing the transcriptome spectrum of individual purified erythroid progenitors isolated from the BM of DBA patients at single-cell resolution, we extended insights into DBA pathogenesis by linking lineage differentiation, translational apparatus and the cell cycle process. In addition, we determined that the therapeutic effect of GCs is exerted through stimulation of the IFN signaling pathway. This finding provides a foundation for the development of novel and more effective pharmacological strategies to treat patients with DBA.

## Materials and methods

### Isolation of BM cells from patients

Cryopreserved BM cells were collected from healthy individuals and DBA patients after informed consent was given, and the present study was approved by the Ethical Committee on Medical Research at the Institute of Hematology and Blood Diseases Hospital (Tianjin, China, KT2019090-EC-2, KT2019090-EC-3). DBA was diagnosed according to World Health Organization criteria. Clinical and laboratory features of the patients were assessed and tabulated in Supplementary Table S1. BM mononuclear cells (BMMNC) were isolated using HISTOPAQUE^®^ density gradient centrifugation (Sigma-Aldrich; Merck KGaA, Darmstadt, Germany, cat no. 10771).

### FACS analysis and sorting of erythroid progenitors

Fluorescence activated cell sorting (FACS) staining was performed as described previously^81^. BMMNC CD45^+^ cells were isolated using microbeads (Miltenyi Biotec, Bergisch Gladbach, Germany, cat. no. 130-045-801) prior to labeling with PE-CD45 (BD Biosciences, Franklin Lakes, NJ, USA, cat. no. 555483), eFluor450-CD3 (eBioscience, Inc., San Diego, CA, USA, cat. no. 48-0037-41), eFluor450-CD4 (eBioscience, Inc., cat. no. 48-0049-41), eFluor450-CD14 (eBioscience, Inc., cat. no. 48-0149-41), eFluor450-CD19 (eBioscience, Inc., cat. no. 48-0199-41), APC-CD41 (BioLegend, San Diego, CA, USA, cat. no. 303710), APC-CD235a (GPA) (BD Biosciences, cat. no. 551336), BV605-CD123 (IL-3R) (BD Biosciences, cat. no. 564197), PerCP-Cy5.5-CD36 (BD Biosciences, cat. 561536), PE-Cy7-CD34 (eBioscience, Inc., cat. 25-0349-42) and FITC-CD71 (eBioscience, Inc., cat. 11-0719-42) antibodies for 30 min at 4 °C in the dark. After washing, BFU-E and CFU-E cells, which were immunophenotyped as CD45^+^CD3^−^CD4^−^CD14^−^CD19^−^CD41^−^CD235a^−^CD123^−^CD36^−^CD34^+^ and CD45^+^CD3^−^CD4^−^CD14^−^CD19^−^CD41^−^CD235a^−^CD123^−^CD36^+^CD71^high^, respectively, were sorted using a FACS instrument (Aria III, BD Biosciences). Data were analyzed using FlowJo software (version 7.6.1, FlowJo, LLC, Ashland, OR, USA).

### Colony forming unit (CFU) assays

Sorted BFU-E cells were seeded into methylcellulose (STEMCELL Technologies, Vancouver, Canada, cat. H4435) following the manufacturer’s protocol. Colonies were cultured at 37 °C in a humidified atmosphere with 5% CO_2_ and assessed on day 14.

### Preparation of scRNA-seq library

Single-cell RNA-seq (scRNA-seq) library preparation was performed as previously reported^28,82,83^. In brief, single cells were obtained via mouth pipetting from sorted bulk BFU-E and CFU-E cells. After reverse transcription (Invitrogen; Thermo Fisher Scientific, Inc., Waltham, MA, USA, cat. 18064-071) and PCR amplification (18 cycles, Kapa Biosystems, Wilmington, MA, USA, cat. kk2602), a 3 μL aliquot from each single cell cDNA sample was pooled and purified using a DNA Clean & Concentrator^™^ kit (Zymo Research Corporation, Irvine, CA, USA, cat. D4014) and AMPure XP Beads (Beckman Coulter, Brea, CA, USA, cat. A63882). Library preparation was performed using KAPA Hyper Prep kits with PCR Library Amplification/Illumina series (Kapa Biosystems, cat. KK8504) and NEBNext^®^ Multiplex Oligos for Illumina^®^ (New England Biolabs, Ipswich, MA, USA, cat. E7335L) after biotin-indexing PCR.

### Preprocessing of single-cell RNA-seq data

Raw sequencing data was demultiplexed based on the cell-specific barcode sequences in read2 of paired-end reads. UMI sequences were extracted from read2 and assigned to paired read1 sequences. The template switch oligo, polyA tail, adaptor, and low-quality sequence (*N* > 10%) were trimmed using Trim Galore (version 0.4.4_dev; http://www.bioinformatics.babraham.ac.uk/projects/trim_galore). The clean reads were aligned to the human reference genome (GRCh38) using STAR^84^ (version 2.5.3a) with the known gene annotation (Gencode v27). Gene expression levels were quantified based on the UMIs after removing duplicated reads using HTSeq^85^ (version 0.9.1).

### Removing batch effects, dimensional reduction and clustering analysis

To rule out potential batch effects, we applied dual biological and analytical batch control strategies. In brief, 16 BFU-E cells purified from the same donor were incorporated in every 96-well plate during library construction, serving as an ideal biological batch control. During data analysis, the sequencing data generated from libraries containing cells from the same donors were visualized by t-SNE, and the data were retained for further analysis if the distribution of control cells did not exhibit confounded experimental batches.

The Seurat^86^ (version 2.3) package was applied to analyze the single cell data. To obtain high quality scRNA-seq data, we used the following criteria: only genes with expression (UMI ≥ 1) detected in >3 cells, and cells with more than 1000 detected genes were retained; samples with ≥10,000 genes detected were excluded to eliminate possible contamination of two or multiple cells. After applying quality control metrics, we generated a matrix containing 1392 cells and 14,839 genes. For normal controls, we first found 422 highly variable genes. To define cell clusters, we performed dimension reduction for each sample by using canonical correlation analysis (CCA). MetageneBicorPlot was used to select CCA dimensions and top20 dimensions were chosen for subspaces alignment among samples. We next used FindClusters (reduction.type = “cca.aligned”, resolution = 1.0, dims.use = 1:9) to define cell clusters. For integrative analysis of all NC and patient data, 1168 highly variable genes and top 20 dimensions for align subspaces and 9 dimensions for RunTSNE were used, respectively.

### Estimation of cellular components and gene set enrichment analysis

We compared the ratio of observed to expected cell numbers in each cell cluster to estimate to cellular composition alterations among sample groups by using the Chi-square test^28^. If Ro/e < 1, we assumed that one cell cluster was decreased in a specific group. If Ro/e > 1, we assumed that one cluster was increased in a specific group. To identify differences in transcription activity for selected gene features between different groups of samples, GSEA^87^ was performed. All gene sets used for GSEA analysis were referred to GO terms.

### Differentially expressed genes and GO enrichment

The FindAllMarkers function in the Seurat package was used to identify cluster-specific marker genes, while the FindMarkers function was applied to identify DEGs between any two given groups. The DEGs with adjusted *P* value (*P* value_adj) < 0.05 and expression fold change (avg_FC) ≥ 1.3 were retained. Enrichr^88^ was used to perform GO enrichment analysis.

### Inducing erythroid differentiation in vitro

Human umbilical CB was collected after obtaining informed consent at the Institute of Hematology and Blood Diseases Hospital (Tianjin, China). Erythroid differentiation was induced as previously described^81^. Briefly, CD34^+^ cells were enriched from CB MNCs using microbeads (Miltenyi Biotec, cat. 130-046-702), and then induced to differentiate using a two-phase culture system. During the 1st phase, CD34^+^ cells were expanded in StemSpan SFEM (STEMCELL Technologies, cat. 09650) supplemented with 100 ng/mL human stem cell factor (SCF) (PeproTech, Rocky Hill, NJ, USA, cat. 300-07), 100 ng/mL human thrombopoietin (PeproTech, cat. 300-18), 100 ng/mL human FMS-like tyrosine kinase 3 ligand (Flt3L) (PeproTech, cat. 300-19-100) and 100 ng/mL human interleukin-6 (IL-6) (PeproTech, cat. 200-06) for 4–5 days. During the 2nd phase, the cells were cultured with 5 ng/mL recombinant human interleukin-3 (IL-3) (Sigma-Aldrich; Merck KGaA, cat. I1646), 100 ng/mL recombinant human SCF (ProSpec-Tany TechnoGene, Ltd., Rehovot, Israel, cat. CYT-255) and 3 IU/mL recombinant human Erythropoietin alfa (EPO) (PeproTech, cat. 100-64) with or without 1 μM dexamethasone (Dex) (Sigma-Aldrich; Merck KGaA, cat. D2915) from day 0 to 8. SCF was supplemented until day 14, and EPO alone was added until the end of the culture period. For IFNα treatment, the cells were treated with IFNα (Beijing Kawin Technology Share-holding Co., Ltd, Beijing, China, cat. S20030030) with or without Dex from day 0 to 11 during the 2nd phase of culture.

For lentiviral infection, the short hairpin RNA (shRNA) sequences for *RPS19* (Supplementary Table S7) were cloned and packed into lentiviral shRNA expression vector pSIH1-H1-copGFP (provided by Jia Yu, Basic Institute of Chinese Academy of Medical Sciences) as previously described^89^. The shRNA sequences for *P53* were cloned and packed into pLKO.1 expression vectors. After 4 days of cell proliferation in phase ǀ, the cells were virally infected with a 50–70% infection efficiency. At day 4 or 18 of differentiation, lentivirus-infected cells were processed for CFSE, apoptosis and DNA damage assays, as described below. Lentiviral-infected GFP^+^ cells were also sorted for real time-quantitative polymerase chain reaction (RT-qPCR) and Western blot analyses.

### RNA extraction and RT-qPCR

At day 4 of the 2nd phase of erythroid differentiation, GFP^+^ cells were sorted and total RNA was extracted using TRIzol (Invitrogen; Thermo Fisher Scientific, Inc., cat. 15596026). cDNA was synthesized using TransScript^®^ II One-Step gDNA Removal and cDNA Synthesis SuperMix kits (TransGen Biotech Co., Ltd., Beijing, China, cat. AH311-02). RT-qPCR was performed using PowerUp^™^ SYBR^™^ Green Master Mix (Applied Biosystems; Thermo Fisher Scientific, Inc., cat. A25742). The 2^(-Delta Delta C(T))^ method is used for quantification of the relative gene expression. The primers are listed in Supplementary Table S7.

### Western blot analysis

At day 4 or 8 of 2nd phase of erythroid differentiation, sorted GFP^+^ cells or the puromycin-selected cells were lysed in Laemmli sample buffer (BioRad, cat. no. 161-0737) and subjected to sodium dodecyl sulfate polyacrylamide gel electrophoresis, as previously described^81^. Primary antibodies used in this study included anti-RPS19 (1:1000, Santa Cruz, cat. sc-100836), anti-P53 (1:500, Santa Cruz, cat. sc-126) or anti-α-tubulin (1:10,000, Abcam, cat. ab7291). The secondary antibodies were horseradish peroxidase-conjugated antibodies. Signals were detected using an ECL kit (Invitrogen, cat. WP20005).

### Apoptosis analysis

On day 4 of differentiation, lentiviral infected-erythroid cells were stained with PE-Annexin V and 7-AAD with 1 × binding buffer (BD Biosciences, cat. 559763) for 15 min on ice in the dark, and then analyzed immediately by FACS using a LSR II instrument (BD Biosciences). Apoptosis levels were analyzed in the GFP^+^ population using FlowJo software (version 7.6.1, FlowJo, LLC).

### Cell cycle analysis

The Seurat^86^ package was used to analyze the cell cycle state of single cells. The CellcycleScore function uses gene sets to calculate the cell cycle score based on the expression values for each single cell. According to this score, single cells were classified as being in G1, S or G2/M phase.

### DNA damage assay

Cytospin slides were prepared using lentiviral infected-erythroid cells at day 4 of differentiation and were fixed in 4% paraformaldehyde (Biosharp Life Sciences, Hefei, China, cat. BL539A) in PBS for 15 min at room temperature, followed by permeabilization using 0.3% Triton-X100 (Solarbio, cat. T8200) (v/v) in PBS for 40 min. Next, the slides were blocked with 2% bovine serum albumin (BSA, Beijing Solarbio Science & Technology Co., Ltd, Beijing, China, cat. A8020) (v/v) and incubated with recombinant anti-γH2AX (1:100, Abcam, Cambridge, UK, cat. ab81299) in PBS containing 1% BSA (v/v) for 1 h at room temperature. This was followed by incubation with Alexa Fluor 594-conjugated secondary antibodies (1:100, Invitrogen; Thermo Fisher Scientific, Inc., cat. A-21207) and Hoechst 33342 staining (1:100, Beyotime Institute of Biotechnology, Haimen, China, cat. C1029). Finally, the slides were cover slipped, examined and photographed using a fluorescence microscope (PerkinElmer, Waltham, MA, USA). At least 200 cells were examined.

### Proliferation score

We used the sum of expression levels of known proliferation-related genes as the proliferation score. The curated proliferation gene set outlined in Giladi et al.^56^ was used in the present study.

### CFSE staining

Erythroid cells were harvested and incubated in 10 μM Cell Trace^™^ Far Red Cell stain (Invitrogen, cat. C34564) for 15 min, and then transferred into the differentiation media for 30 min at 37 °C in the dark. Next, cells were seeded into 24-well plates and cultured for an additional 48 h before fixation with 4% paraformaldehyde. Finally, GFP^+^ cells were analyzed by FACS using an LSR II instrument (BD Biosciences).

### MTS assay

Cell proliferation was measured using MTS assays. During the 2nd phase of erythroid differentiation, cells were seeded into a 96-well plate at a density of 5 × 10^4^/mL, and treated with Dex (1 μM) alone, IFNα (1, 10, 100, 1000 IU/mL) alone, or both reagents. On day 3–5, 20 μL MTS reagent (Promega Corporation, Madison, WI, USA, cat. G3581) was added to each well. After 2 h incubation at 37 °C in the dark, absorbance readings were taken at wavelength 490 nm using a microplate reader (BioTek Instruments; Agilent Technologies, Inc., Santa Clara, CA, USA).

### Statistical analysis

Groups were compared using Student’s *t* tests. For scRNA-seq data analysis, *P* value adjustment was performed using the Bonferroni correction when we used the Seurat FindMarkers function to identify DEGs or the FindAllMarkers function for the identification of cell type-specific marker genes. Wilcoxon rank-sum tests were applied to estimate the transcriptional difference of specified gene sets, and *P* values were corrected using the ‘Holm’ method (R package ‘ggpubr’). *P* ≤ 0.05 was considered to indicate a statistically significant difference, and significant *P* values are indicated with asterisks in the figures. Normalized enrichment score (NES) and false discovery rate were used for GSEA analysis. Other analyses and visualizations were performed using GraphPad Prism or R packages.

## Supplementary information

Supplementary Information

Supplementary Table S1

## Data Availability

The accession number for the sequencing data reported in this paper is GSA: HRA001111.
