# Supplementary material for: Decoding the pathogenesis of Diamond–Blackfan anemia using single-cell RNA-seq
Source: Cell Discov. 2022 May 10;8:41. doi: 10.1038/s41421-022-00389-z (PMC9085895; doi:10.1038/s41421-022-00389-z)
Supplement: Supplementary file 1 — Supplementary Information [file 41421_2022_389_MOESM1_ESM.pdf]

Supplementary Fig. S1

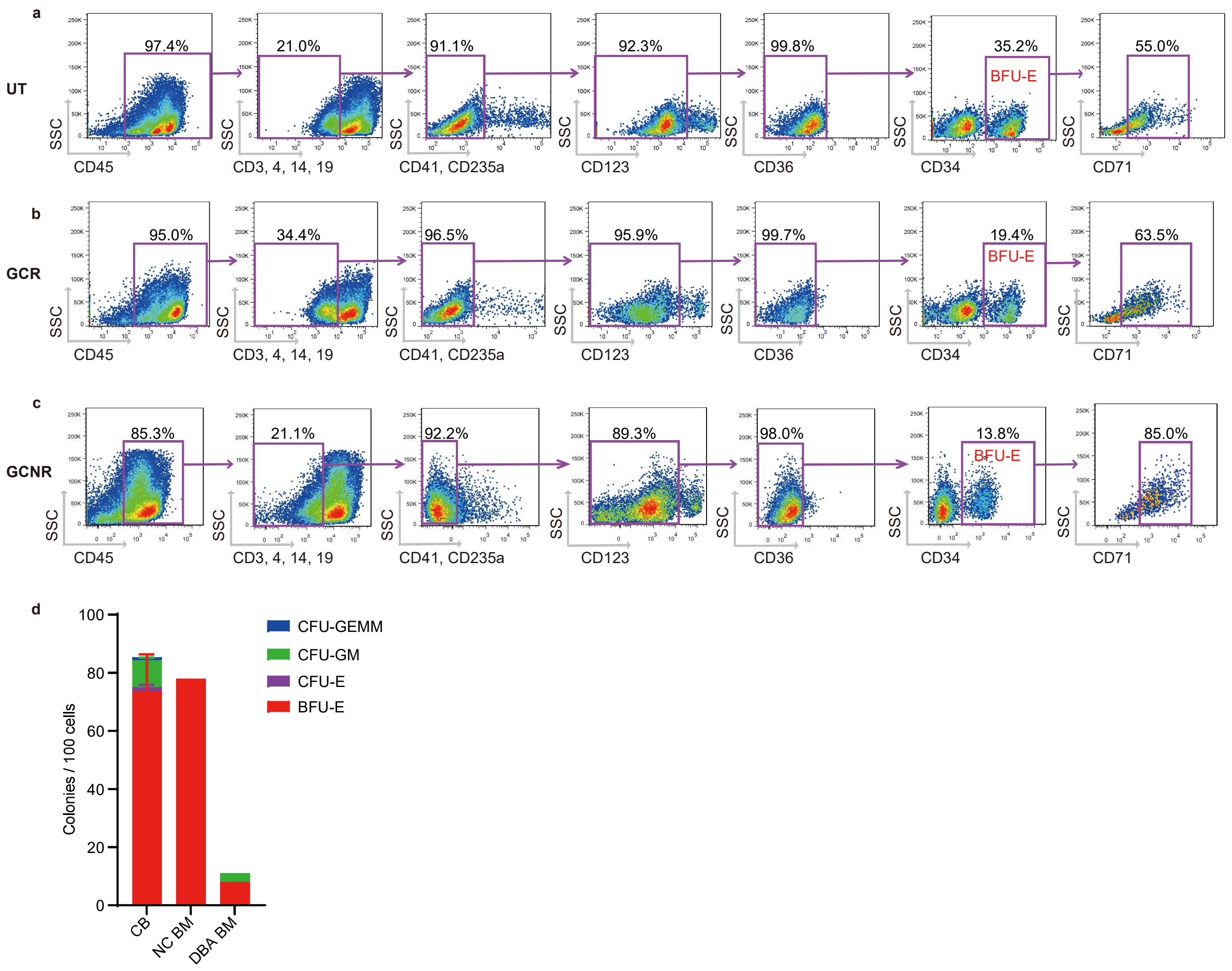

Supplementary Fig. S1 FACS strategy to sort BFU-E cells from DBA patients. a-c Representative FACS plots showing the gating strategy used for the isolation of BFU-E cells ($CD45^{+}CD3^{-}CD4^{-}CD14^{-}CD19^{-}CD41^{-}CD235a^{-}CD123^{-}CD36^{-}CD34^{+}$) from DBA patients from the UT (a), GCR (b) and GCNR (c) group. **d** The number of colonies per 100 FACS-sorted BFU-E cells, which were isolated from cord blood (CB), NC BM and DBA BM mononuclear cells, respectively. We have seeded the sorted cells from three CB samples, one NC BM and one DBA BM sample due to the limited BM samples.

Supplementary Fig. S2

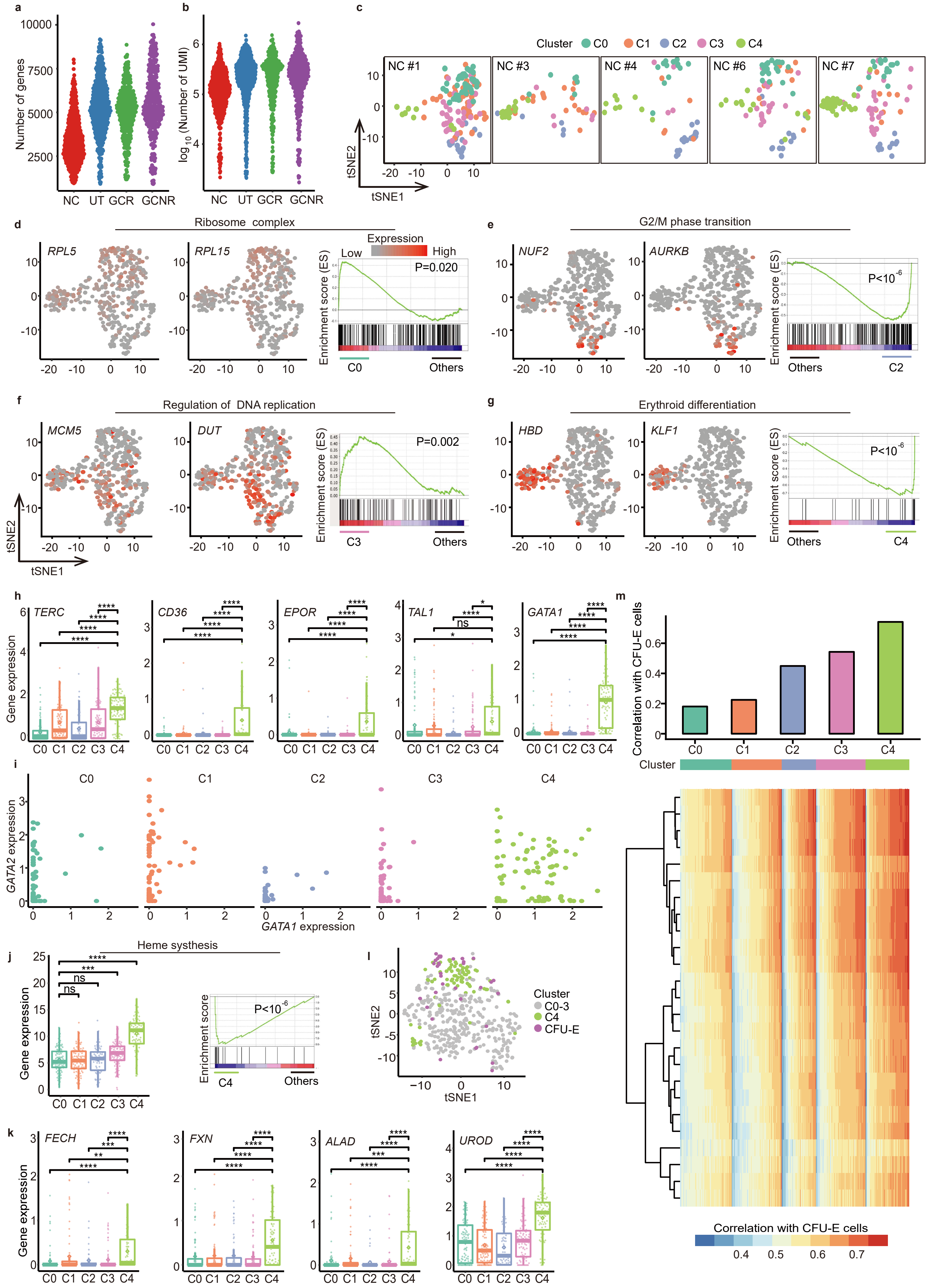

Supplementary Fig. S2 Characterization of erythroid progenitors in normal individuals (NC) by scRNA-seq. a, b Dot plots showing the number of genes (a) and the number of unique molecular identifiers (UMI) (b) detected in each cell among the different groups. **c** t-SNE plots showing cell clusters of each individual NC. **d-g** The red dots on t-SNE plots show the representative signature genes in C0 (*RPL5* and *RPL15*) (d), C2 (*NUF2* and *AURKB*) (e), C3 (*MCM5* and *DUT*) (f) and C4 (*HBD* and *KLF1*) (g), respectively. GSEA plots show the significant enrichment of ribosomes in C0 (d), G2/M phase transition in C2 (e), regulation of DNA replication in C3 (f) and erythroid differentiation in C4 (g) when compared with other clusters. **h** Beeswarm plots showing the expression of the indicted key erythroid effectors or regulators across clusters. **i** *GATA1* (x-axis) and *GATA2* (y-axis) expression across C0-C4 clusters. **j** Beeswarm plot (left) showing the expression levels of the heme biosynthetic process across clusters. Each dot represents the expression value for each single cell that was calculated by summing the $\log_2$ transformed UMI of every gene within the gene set. The GSEA plot (right) shows the enrichment of the heme biosynthetic process in C4. **k** The expression of indicated genes involved in the heme biosynthetic process across clusters. **l** t-SNE plot illuminating the subsets of C4 (green) and CFU-E (purple) among all erythroid progenitors in the NC group. **m** Bar graph (upper) and heatmap (lower) showing the correlation between CFU-E and each BFU-E cluster. In all beeswarm plots of h, j and k, diamonds represent the mean expression value for each cluster, and

boxes represent the median and quartiles. P-values were determined via Wilcoxon rank sum tests. **** $P \leq 0.0001$, *** $P \leq 0.001$, ** $P \leq 0.01$ and * $P \leq 0.05$; with ns denoting no significance.

Supplementary Fig. S3

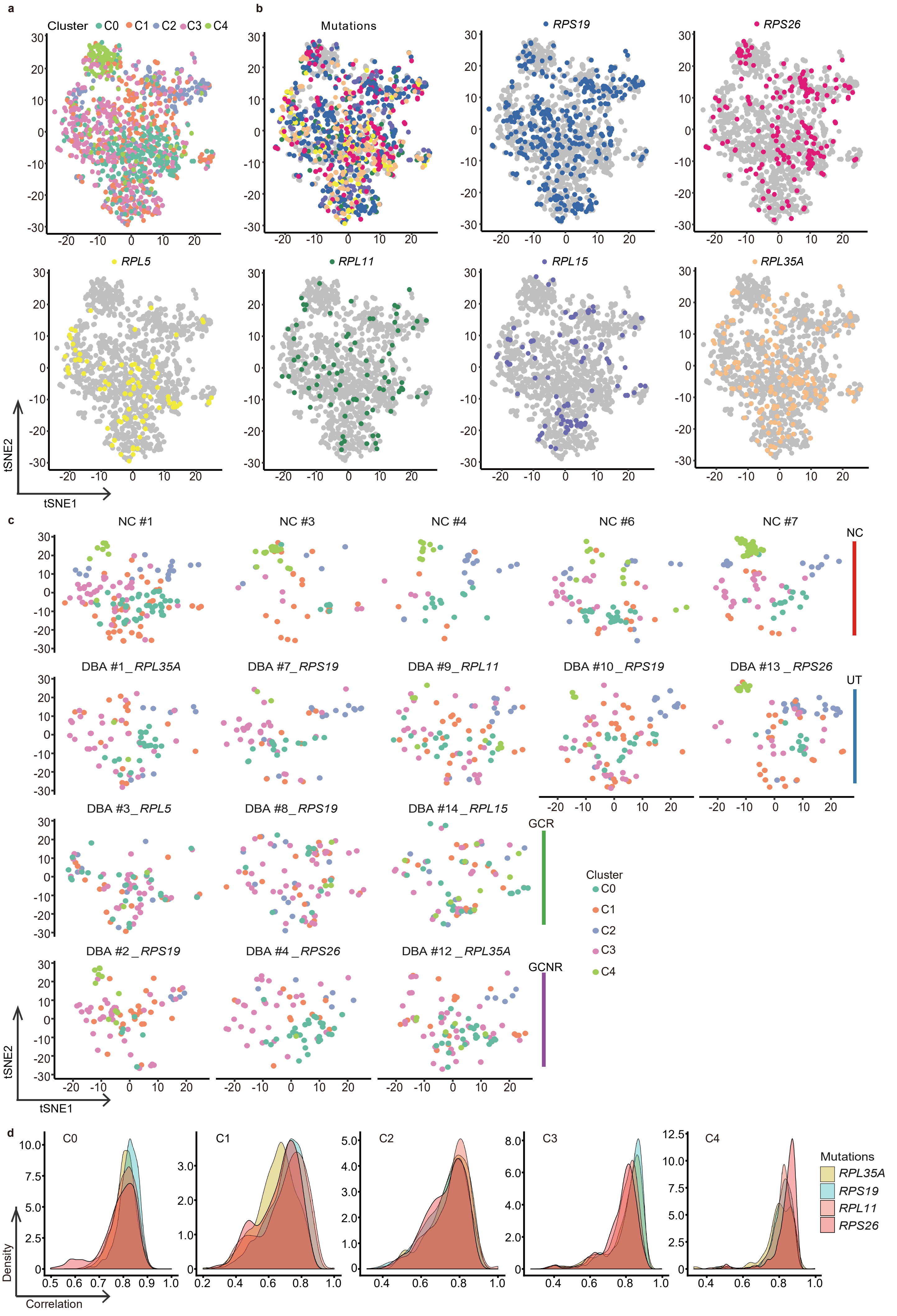

Supplementary Fig. S3 Characterization of erythroid progenitors in DBA patients. a t-SNE plot showing cell clusters by integrating all cells from each individual sample. **b** Cells with distinct gene mutations are color-coded and shown in the t-SNE plots. **c** t-SNE plots showing cell clusters from each individual sample. **d** Density plot indicating the level of transcriptomic correlation among mutations within each cluster in UT patients.

Supplementary Fig. S4

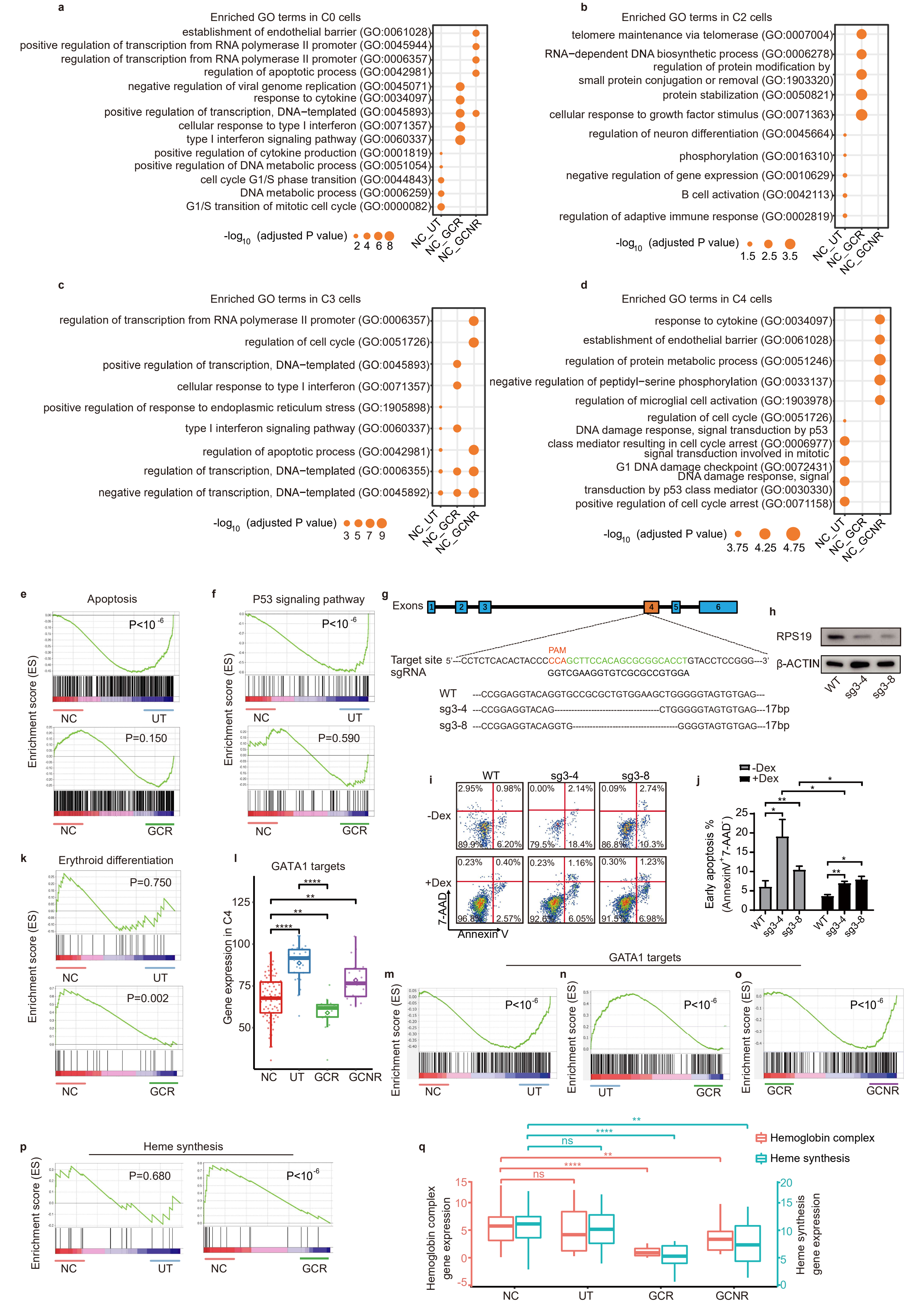

Supplementary Fig. S4 P53 signaling and apoptosis are increased in C4 of DBA untreated cells. a-d The enriched GO terms of differentially expressed genes in C0 (a), C2 (b), C3 (c) and C4 (d) between the NC and UT, the NC and GCR and the NC and GCNR groups. The top 5 enriched GO terms are presented after integrating terms belonging to similar categories. The size of the dots represents the $-\log_{10}$(adjusted P value). **e, f** GSEA plots showing the significant enrichment of genes associated with apoptosis (e) and P53 signaling pathway (f) in UT C4 cells when comparing NC and UT (upper panel). Whereas no significant differences were detected among these genes in GCR C4 cells when comparing NC and GCR groups (bottom panel). **g, h** Generation of *RPS19+/-* cell lines using the CRISPR/Cas9 technology in HUDEP2 cells. A schematic illustrating the experimental design. Exon 4 of RPS19 is targeted by sgRNAs and the nucleotide sequences in WT and RPS19 heterozygous depleted cell lines targeted by sgRNAs (sg3-4 and sg3-8) (g). The protein expression of RPS19 upon deletion, measured Western blotting (h). **i, j** The representative FACS plot (i) and bar graph (j) showing apoptosis level (measured by the percentage of Annexin V^{+}7-AAD^{-} cells) in *RPS19*$^{+/-}$ HUDEP-2 cells cultured with or without Dex. Results are presented as the mean ± SEM, and P-values were determined using Student's t-tests $^{**}P<0.01$ and $^{*}P<0.05$; $n \geq 3$ independent experiments. **k** GSEA plots showing the enrichment of erythroid differentiation in C4, comparing NC with UT (upper panel) and NC with GCR (bottom panel). **l** The expression level of the GATA1 targets in erythroid

cells [20] in C4 across groups. Each dot represents the expression value for each single cell that was calculated by summing the $\log_2$ transformed UMI of every gene within the gene set. Diamonds represent the mean expression value for each cluster, and the boxes represent median and quartiles. P-values were determined by Wilcoxon rank sum test. ****P≤0.0001 and **P≤0.01. **m-o** GSEA plots showing the enrichment of GATA1 targets in C4, comparing NC and UT (m), UT and GCR (n) and GCR and GCNR (o). **p** GSEA plots showing the enrichment of heme synthesis in C4, comparing NC with UT (left) and NC with GCR (right). **q** The expression of hemoglobin complex (left y-axis, red) and heme synthesis (right y-axis, blue) in C4 in each group. The expression of indicated genes sets was calculated by summing the $\log_2$ transformed UMI of every gene within the gene set. Boxes represent the median and quartiles. P-values were determined using Wilcoxon rank sum tests. ****P≤0.0001 and **P≤0.01.

Supplementary Fig. S5

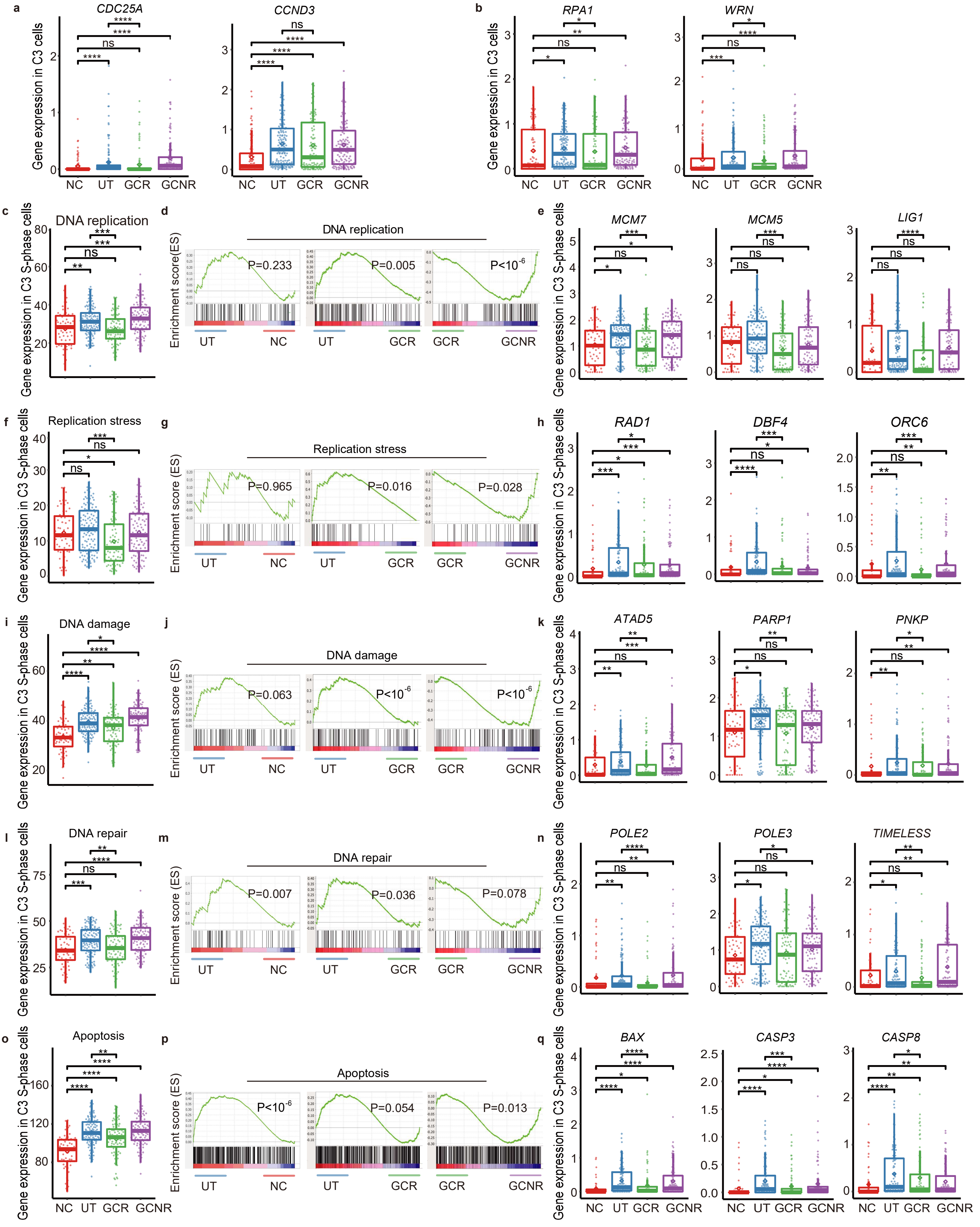

Supplementary Fig. S5 DNA replication stress is reduced in GCR C3 cells during S-phase. a, b Beeswarm plots showing the expression of G1/S transition-related genes (*CDC25A* and *CCND3*) (a) and DNA replication-related genes (*RPA1* and *WRN*) (b) in C3 in the indicated groups. **c, f, i, l and o** Plots showing the expression level of gene sets associated with DNA replication (c), DNA replication stress (f), DNA damage (i), DNA repair (l) and apoptosis (o) in C3 cells during S-phase of the cell cycle among the indicated groups. **d, g, j, m and p** GSEA plots showing the enrichment of genes associated with the regulation of DNA replication (d), DNA replication stress (g), DNA damage (j), DNA repair (m) and apoptosis (p) in C3 cells during the S-phase of cell cycle, with comparisons made between the UT and NC, the UT and GCR and the GCR and GCNR groups. **e, h, k, n and q** Beeswarm plots showing the expression of indicated genes associated with DNA replication (*MCM7*, *MCM5* and *LIG1*) (e), DNA replication stress (*RAD1*, *DBF4*, and *ORC6*) (h), DNA damage (*ATAD5*, *RAPP1* and *PNKP*) (k), DNA repair (*POLE2*, *POLE3* and *TIMELESS*) (n) and apoptosis (*BAX, CASP3* and *CASP8*) (q) in C3 cells during the S-phase of the cell cycle among the indicated groups. Each dot represents the expression value for each single cell that was calculated by summing the $\log_2$ transformed UMI of every gene within the gene set. Diamonds represent the mean expression value for each cluster, and boxes represent the median and quartiles. P-values were determined using Wilcoxon rank sum tests unless otherwise indicated. ****P≤0.0001; ***P≤0.001; **P≤0.01 and *P≤0.05; with ns

denoting no significance.

Supplementary Fig. S6

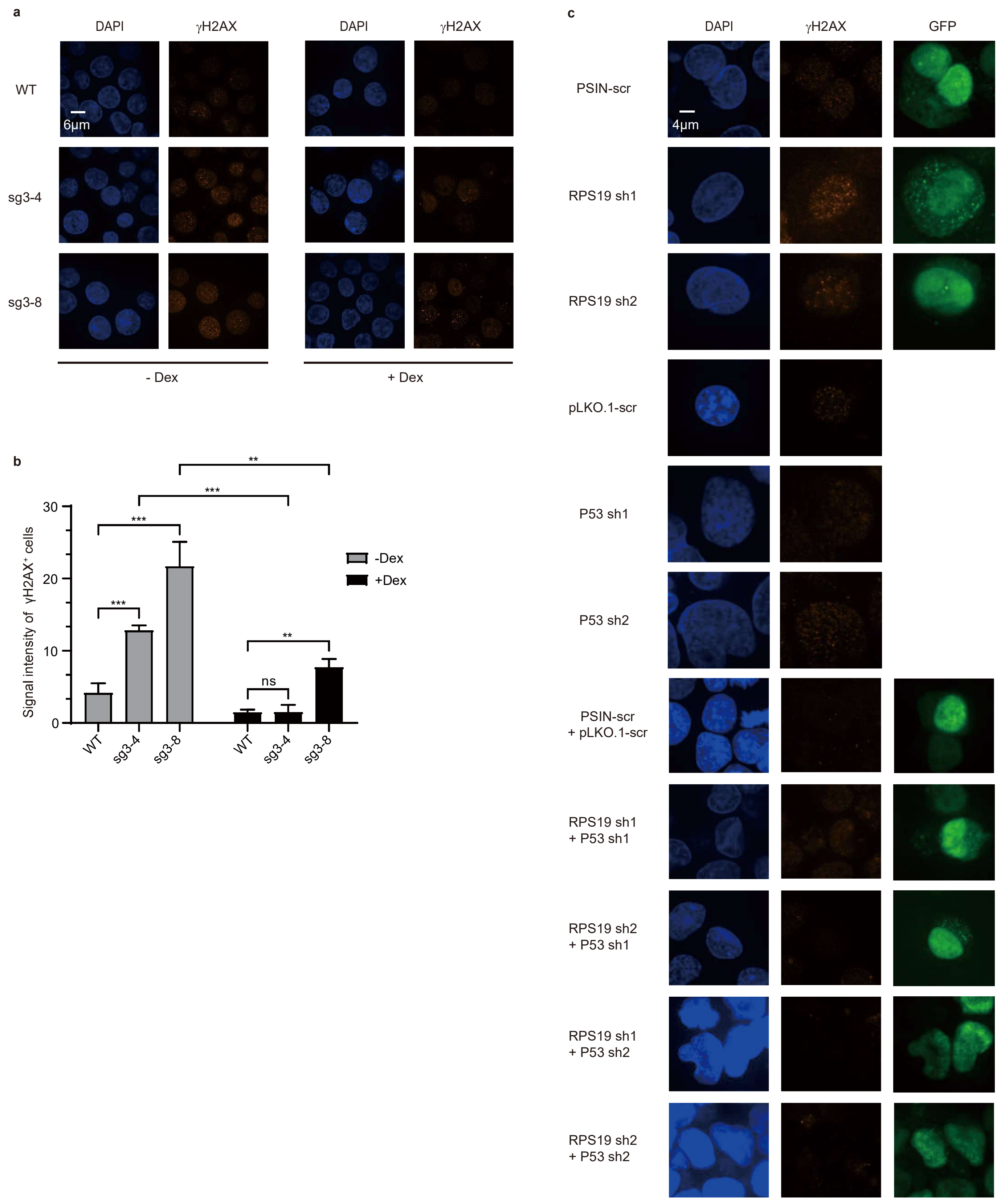

Supplementary Fig. S6 Immunofluorescence staining of DNA damage. a Immunofluorescence image of DNA damage, indicated by γH2AX expression, in *RPS19*$^{+/-}$ HUDEP-2 cells with or without Dex treatment. Scale bar = 6 μm. **b** Quantification of immunofluorescent foci of γH2AX. More than 200 cells were analyzed. Results are presented as the mean ± SEM, and P-values were determined using Student's t-tests. ***P<0.001 and **P<0.01; with ns denoting no statistical significance. n=3 independent experiments. **c** Immunofluorescence showing DNA damage through measuring γH2AX expression in erythroid cells induced from CB-CD34^{+} HSPCs, on day 8 of differentiation. CD34^{+} HSPCs were infected either with *RPS19* shRNAs expressing GFP alone, *P53* shRNAs expressing puromycin alone, or both shRNAs simultaneously after 4 days of phase I proliferation. After 8 days of erythroid differentiation, cells were collected for immunofluorescence staining. Scale bar = 4 μm.

Supplementary Fig. S7

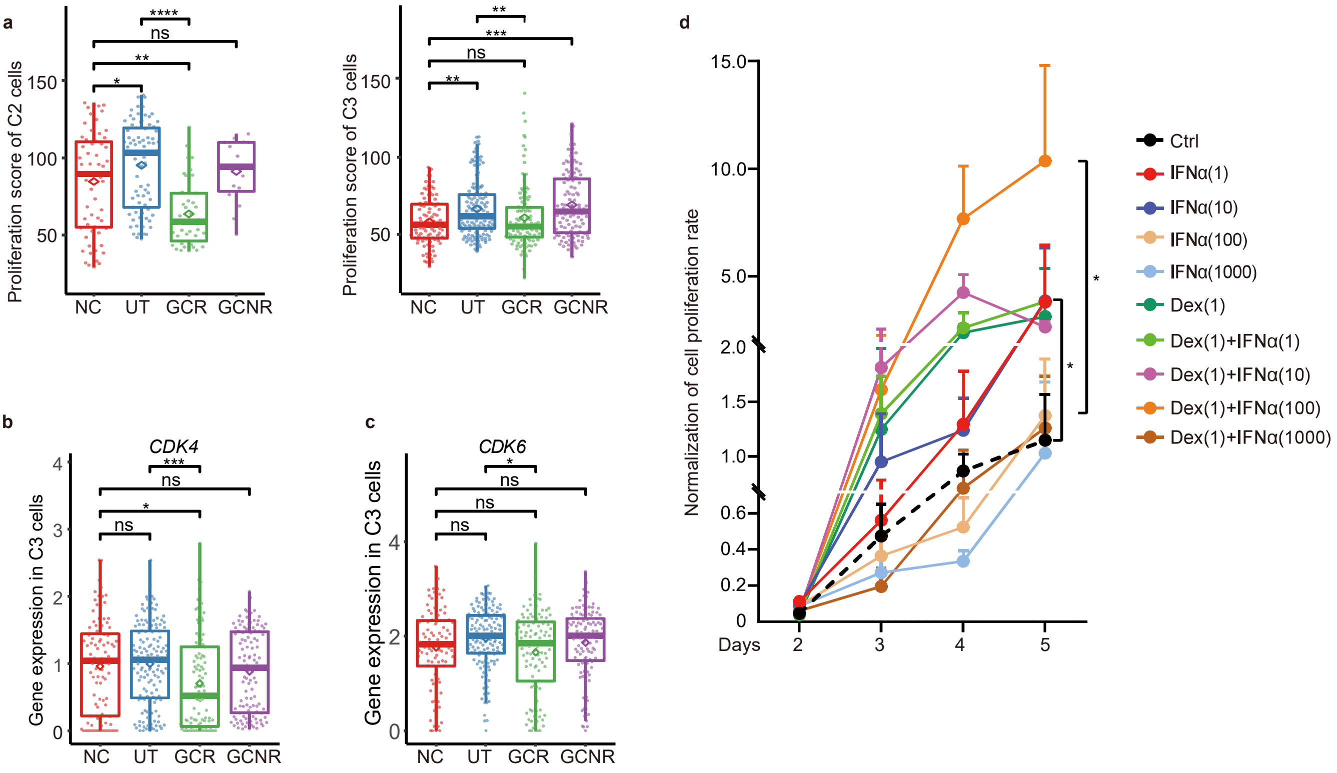

Supplementary Fig. S7 Elevated IFN signaling in GCR C3 cells. a Expression of a set of proliferation signatures[57] in C2 (left) and C3 (right) cells across the indicated groups. **b, c** Boxplots showing the expression of cell cycle regulators *CDK4* (b) and *CDK6* (c) in C3 cells among different groups. **d** Time course proliferation of *RPS19*-depleted, cord blood-derived erythroid cells during erythroid differentiation when treated either with IFNα alone, Dex alone, or a combination of both. Results are presented as the mean ± SEM and P-values were determined using Student's t-tests. *P<0.05; with ns denoting no statistical significance. n=3 independent experiments. In the beeswarm plots (a, b and c), diamonds represent the mean expression value for each cluster, and boxes represent the median and quartiles. P-values were determined using Wilcoxon rank sum tests. ****P≤0.0001, ***P≤0.001, **P≤0.01 and *P≤0.05; with ns denoting no significance.

Supplementary materials and methods

HUDEP-2 cell culture

Human umbilical cord blood-derived erythroid progenitor cells (HUDEP-2), obtained from RIKEN BioResource Research Center (Kyoto, Japan), were cultured in StemSpan SFEM® medium, in the presence of SCF (50 ng/mL), EPO (3 IU/ mL), doxycycline (1 μg/mL) (Sigma-Aldrich; Merck KGaA, cat. no. 9891), dexamethasone (10^{-6} M) and 1% penicillin and streptomycin.
